# Awareness Is Bliss: How Acquiescence Affects Exploratory Factor Analysis

**DOI:** 10.1177/00131644221089857

**Published:** 2022-05-16

**Authors:** E. Damiano D’Urso, Jesper Tijmstra, Jeroen K. Vermunt, Kim De Roover

**Affiliations:** 1 Tilburg University, The Netherlands

**Keywords:** response styles, ARS (acquiescence response style), EFA (exploratory factor analysis), response bias

## Abstract

Assessing the measurement model (MM) of self-report scales is crucial to obtain valid measurements of individuals’ latent psychological constructs. This entails evaluating the number of measured constructs and determining which construct is measured by which item. Exploratory factor analysis (EFA) is the most-used method to evaluate these psychometric properties, where the number of measured constructs (i.e., factors) is assessed, and, afterward, rotational freedom is resolved to interpret these factors. This study assessed the effects of an acquiescence response style (ARS) on EFA for unidimensional and multidimensional (un)balanced scales. Specifically, we evaluated (a) whether ARS is captured as an additional factor, (b) the effect of different rotation approaches on the content and ARS factors recovery, and (c) the effect of extracting the additional ARS factor on the recovery of factor loadings. ARS was often captured as an additional factor in balanced scales when it was strong. For these scales, ignoring extracting this additional ARS factor, or rotating to simple structure when extracting it, harmed the recovery of the original MM by introducing bias in loadings and cross-loadings. These issues were avoided by using informed rotation approaches (i.e., target rotation), where (part of) the rotation target is specified according to a priori expectations on the MM. Not extracting the additional ARS factor did not affect the loading recovery in unbalanced scales. Researchers should consider the potential presence of ARS when assessing the psychometric properties of balanced scales and use informed rotation approaches when suspecting that an additional factor is an ARS factor.

## Introduction

Evaluating the psychometric properties of self-report scales in behavioral sciences is crucial for a valid assessment of individuals’ latent constructs (e.g., self-esteem). Commonly, the assessment of these psychometric properties entails, among other things, evaluating the measurement model (MM). The latter indicates how many latent constructs or factors are measured by the items, and which factor is measured by which items. Also, it needs to be determined whether items are good measurements of latent constructs (i.e., how strongly they load on factors), and whether they measure more than one latent construct at the same time (i.e., load on multiple factors).

The most frequently used method to unravel the psychometric properties of newly developed scales is EFA. Without imposing an assumed structure on the factor loadings, except (possibly) the number of factors, EFA identifies the relations between factors and items by analyzing the item correlations. Because of its advantageous exploratory nature as well as its popularity, EFA is often considered a mandatory step in the context of scale construction (Goretzko et al., 2021; Howard, 2016).

An important limitation of self-report scales is that, despite their widespread use, they might not always sufficiently capture the psychological trait being measured (Van Vaerenbergh & Thomas, 2013). In fact, subject responses might not always be consistent with the measured psychological construct (Bolt & Johnson, 2009). These inconsistencies, generally defined as response styles (RSs) or response bias, can be viewed as systematic or stylistic tendencies in the manner respondents use a rating scale when responding to self-report items (Paulhus, 1991). One well-known RS is the acquiescent one, which is a tendency to agree with items regardless of their content (Van Vaerenbergh &Thomas, 2013).

Failing to take into account acquiescence response style (ARS) can harm psychometric analyses in many ways. For instance, ARS can inflate observed means and correlations (Van Vaerenbergh & Thomas, 2013), increase or decrease the strength of relations between factors and items (Ferrando & Lorenzo-Seva, 2010), and result in an additional factor (Billiet & McClendon, 2000). These potential artifacts not only interfere with the psychometric assessment of the properties of a scale but can also invalidate the interpretation of subjects’ scale scores (Bolt & Johnson, 2009).

When the scale has been previously validated, the number of factors to be measured, and their zero-loading structure are known *a priori*. In such cases, ARS can be explicitly included in the MM as an additional factor. Previous research has demonstrated how ARS can be easily incorporated in the context of confirmatory factor analysis (Billiet & McClendon, 2000), item response theory (Falk & Cai, 2016), and latent class analysis (Morren et al., 2011). One crucial limitation of these confirmatory approaches, however, is the need for *a priori* knowledge regarding the MM, which is, of course, lacking when the goal is to determine this MM in the first place.

The assessment of a scale’s MM can, therefore, be difficult when ARS causes distortions. In EFA, the number of factors is usually evaluated and, upon resolving rotational freedom, an additional factor could be erroneously interpreted as a dimension of the psychological construct of interest, while it is merely a consequence of ARS. In addition, when not taking ARS into account in the rotation, items may seem to measure more than one factor at the same time, or seem to be a bad measurement of a factor (i.e., low loading), which might lead researchers to drop these seemingly malfunctioning items from the scale. Furthermore, in the most extreme case in which most, or all, items are heavily affected by ARS, the whole scale may seem to be disfunctional.

While some methods have been proposed to reduce the effects of ARS on EFA (Ferrando et al., 2003, 2016; Lorenzo-Seva & Rodríguez-Fornells, 2006) only a few papers examined the impact of ignoring or being unaware of ARS on the recovery of factor loadings (Ferrando & Lorenzo-Seva, 2010; Savalei & Falk, 2014). The latter studies, however, have mostly dealt with scales measuring only a single content factor (i.e., unidimensional scales) measured by continuous items or items that may be treated as such (i.e., items with more than five categories; Rhemtulla et al., 2012), which only partially mirror the features of commonly used self-report scales and preclude investigating the influence of rotation. In addition, none of these studies investigated to what extent ARS is retrieved as an additional factor by commonly used model selection criteria (e.g., Bayesian Information Criterion; Schwarz, 1978), which, in empirical practice, would generally precede any further investigation of the loadings. Drawing upon these existing gaps in current research, this article aims to extensively study the impact of ARS on the assessment of the psychometric properties of self-report scales, as well as strategies to account for ARS when using EFA. This investigation comprises a simulation study on unidimensional and multidimensional scales for two types of data (i.e., ordinal and approximately continuous data). In addition, we simulated a null scenario (i.e., without an ARS factor) that served as a point of comparison. By means of this simulation study, we will assess (a) how often and in which conditions different model selection criteria retain the additional ARS factor, (b) the effect of different rotation approaches on the recovery of the content and ARS factors when the additional ARS factor is retained, and (c) the effect of (not) retaining the ARS factor on the recovery of the (properly rotated) factor loadings and correlations.

The remainder of the article proceeds as follows: in section “Theoretical Framework,” we provide a general introduction to EFA and how ARS can affect some of its main steps, namely dimensionality assessment and factor rotation. For factor rotation, we discuss two types of rotation, namely rotation to simple structure (i.e., as one usually does when unaware of a potential ARS) and informed rotation approaches (e.g., rotation to a partially specified target that takes the potential ARS factor into account). Section “Simulation Study” focuses on a simulation study that evaluates the performance of EFA in assessing the psychometric properties of unidimensional and multidimensional scales (with and without the presence of ARS). Finally, in section “Discussion,” recommendations are formulated based on the results of the simulation study along with limitations of the current study and future research directions.

## Theoretical Framework

### Factor Analysis Model With ARS

Consider that continuous responses by N subjects on J items are collected in a data matrix X , and that each item response is a measure of the following three common factors: (a) Two intended-to-be-measured (i.e., content) factors $\eta_{1}$ and $\eta_{2}$ and (b) an ARS factor $\eta_{\mathrm{ARS}}$ . A factor analysis model describes the response $x_{\mathrm{ij}}$ of subject i on item j as

(1) $$x_{\mathrm{ij}}=\nu_{j}\;+\;\lambda_{j_{1}}\eta_{i_{1}}\;+\;\lambda_{j_{2}}\eta_{i_{2}}\;+\;\lambda_{j_{\mathrm{ARS}}}\eta_{i_{\mathrm{ARS}}}\;+\;\epsilon_{\mathrm{ij}}$$

where $\nu_{j}$ is an item-specific intercept, $\lambda_{j_{1}}$ , $\lambda_{j_{2}}$ , and $\lambda_{j_{\mathrm{ARS}}}$ are the loadings on item j on the three factors, $\eta_{i_{1}}$ , $\eta_{i_{2}}$ , and $\eta_{i_{\mathrm{ARS}}}$ are the factors scores of subject i , respectively, and $\epsilon_{\mathrm{ij}}$ is the residual. Factors are assumed to be multivariate normally distributed ~ MVN(α,ϕ) ,^1^ independently of ϵ , which are ~ MVN(0,ψ) , with ψ containing the unique variances $\psi_{j}$ on the diagonal and zeros on the off-diagonal.

When using exploratory factor analysis (EFA; Lawley & Maxwell, 1962) as a first step in assessing the psychometric properties of a scale, the factors in (1) are not (yet) labeled (i.e., researchers do not have or impose *a priori* assumptions on whether a factor corresponds to a certain content factor or an ARS). Also, the assumption of continuous item responses often cannot be safely made, especially in the case of ordered-categorical variables (e.g., a Likert-type-scale item with “disagree,”“neither agree nor disagree,” and “agree” as response options). In that case, it is better to assume that the data matrix X is composed of polytomously scored responses that can take on C possible values with c={0,1,2,...,C−1} . In a categorical EFA model, it is assumed that each of the observed responses is obtained from a discretization of a continuous unobserved response variable $x_{\mathrm{ij}}^{*}$ through some thresholds parameters $\tau_{j,c}$ . The threshold parameters indicate the separation between the response categories, where the first and last thresholds are defined as $\tau_{j,0}\;=-\infty$ and $\tau_{j,C}\;=-\infty$ , respectively. In formal terms,

(2) $$x_{\mathrm{ij}}=c,\mathrm{if}\tau_{j,c}\;<\;x_{\mathrm{ij}}^{*}\;<\;\tau_{j,\;c\;+\;1}\;c=0,\;1,\;2,\;...,\;C-1.$$

A categorical EFA model for the vector of scores $x_{i}^{*}$ of subject i can be specified as

(3) $$x_{i}^{*}=\nu^{*}\;+\;\Lambda \eta_{i}\;+\;\epsilon_{i}$$

where $\nu^{*}$ is a J -dimensional vector of latent intercepts (i.e., intercepts of the unobserved response variables in $x_{i}^{*}$ ), Λ is a J × Q matrix of factor loadings, $\eta_{i}$ is a Q -dimensional vector of scores on the Q factors, $\epsilon_{i}$ is a J -dimensional vector of residuals. Gathering the loadings of the unlabeled factors in a matrix Λ , the model implied covariance matrix ∑ is obtained as

(4) ∑=ΛϕΛ+ψ

Polychoric correlations are generally used as the input for categorical EFA, where the correlation between ordinal items is computed as the correlation of the standard bivariate normal distribution of their latent response variables $x_{\mathrm{ij}}^{*}$ (Ekström, 2011). Furthermore, they are known to produce unbiased parameters estimates in factor analysis models (Babakus et al., 1987; Rigdon & Ferguson Jr, 1991), whereas with Pearson correlations, which are commonly used for estimating EFA with continuous item responses, the correlations among ordered-categorical items are commonly underestimated (Bollen & Barb, 1981).

### Potential Effects of ARS on Factor Rotation

Factors obtained from EFA have rotational freedom (i.e., rotating them does not affect model fit; Browne, 2001), which should be resolved to obtain an interpretable solution. Commonly, the goal is to strive for simple structure and different criteria can be applied to minimize the variable complexity (i.e., number of non-zero loadings per variable), the factor complexity (i.e., number of non-zero loadings per factor), or a combination of both (Schmitt & Sass, 2011). In this article, we focus on minimizing the variable complexity by means of oblique simple structure rotation (i.e., allowing the factors to become correlated) because there are little to no theoretical reasons to assume that the content factors are uncorrelated in case of multidimensional constructs and minimizing the variable complexity matches the idea of non-ambiguous items that are clear measurements of only one factor. In addition, this rotation allows content factors and the ARS factor to be correlated, which, according to recent literature, is both theoretically and empirically acceptable for some personality traits (e.g., agreeableness, extraversion, impulsiveness; see Weijters et al., 2010 and Ferrando et al., 2016 for a review).

**Figure 1.**
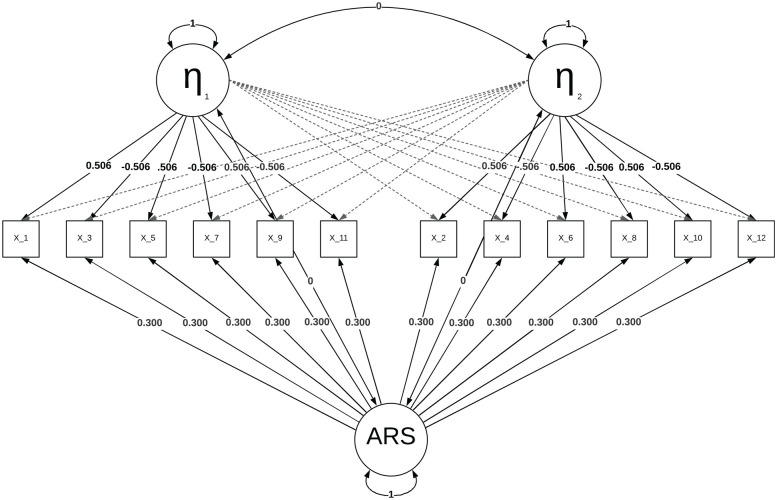
A Multidimensional Factor Model With an ARS Factor *Note.* Where the two content factors are defined as $\eta_{1}$ and $\eta_{2}$ , and ARS stands for the ARS factor. The zero and non-zero loadings are indicated by normal and dashed lines, respectively, and the residuals are omitted for visual clarity. ARS = acquiescence response style.

Simple structure can be pursued with uninformed or informed rotation approaches, where the former applies no *a priori* assumptions on the MM structure and the latter involves rotating to a (partially) specified target based on such *a priori* assumptions. To exemplify how (un)informed simple structure rotation can be affected by the presence of an ARS, we make use of an illustrative example, whose loadings are displayed in Table 1. Specifically, the top part of the table displays the (partially) specified targets for the informed rotation approaches, while the bottom part displays the different sets of rotated loadings. Moreover, the values in the target original matrix were used as the population values of the loadings to generate the data with N = 10,000—implying that the estimated loadings are likely very close to the population values. A visual representation of this model is depicted in Figure 1, where $X_{1}$ – $X_{12}$ represent item responses.^2^

**Table 1.** (Semi-) Specified Targets (Top), and Rotated Loadings Using Uninformed and Informed Rotation Approaches (Bottom) of an EFA Model With 12 Items and Three Factors for an Illustrative Example.

Target matrices												
				Target original			Target			Semi-specified target		
				$\eta_{1}$	$\eta_{2}$	ARS	$\eta_{1}$	$\eta_{2}$	ARS	$\eta_{1}$	$\eta_{2}$	ARS
$X_{1}$				0.506	0	0.295	1	0		NA	0	NA
$X_{2}$				0	0.506	0.295	0	1	1	0	NA	NA
$X_{3}$				−0.506	0	0.295	−1	0	1	NA	0	NA
$X_{4}$				0	−0.506	0.295	0	−1	1	0	NA	NA
$X_{5}$				0.506	0	0.295	1	0	1	NA	0	NA
$X_{6}$				0	0.506	0.295	0	1	1	0	NA	NA
$X_{7}$				−0.506	0	0.295	−1	0	1	NA	0	NA
$X_{8}$				0	−0.506	0.295	0	−1	1	0	NA	NA
$X_{9}$				0.506	0	0.295	1	0	1	NA	0	NA
$X_{10}$				0	0.506	0.295	0	1	1	0	NA	NA
$X_{11}$				−0.506	0	0.295	−1	0	1	NA	0	NA
$X_{12}$				0	−0.506	0.295	0	−1	1	0	NA	NA
Rotated loadings												
	Oblimin			Target original			Target			Semi-specified target		
	$\eta_{1}$	$\eta_{2}$	ARS	$\eta_{1}$	$\eta_{2}$	ARS	$\eta_{1}$	$\eta_{2}$	ARS	$\eta_{1}$	$\eta_{2}$	ARS
$X_{1}$	0.548	−0.071	−0.099	0.483	−0.011	0.294	0.481	−0.008	0.290	0.490	0.010	0.278
$X_{2}$	0.144	0.551	0.126	0.009	0.484	0.280	0.007	0.488	0.288	−0.015	0.485	0.288
$X_{3}$	−0.145	−0.101	0.520	−0.476	.009	0.279	−0.477	0.014	0.283	−0.469	−0.012	0.294
$X_{4}$	0.254	−0.373	0.303	−0.011	−0.480	0.298	−0.013	−0.476	0.290	0.004	−0.478	0.290
$X_{5}$	0.537	−0.068	−0.130	0.497	−0.005	0.265	0.495	−0.002	0.262	0.502	0.003	0.250
$X_{6}$	0.108	0.566	0.123	−0.016	0.506	0.258	−0.018	0.510	0.266	0.011	0.508	0.266
$X_{7}$	−0.145	−0.079	0.520	−0.475	−0.012	0.276	−0.476	−0.007	0.279	−0.468	0.010	0.290
$X_{8}$	0.238	−0.397	0.271	0	−0.493	0.260	−0.001	−0.490	0.252	−0.006	−0.492	0.252
$X_{9}$	0.520	−0.085	−0.120	0.476	0.012	0.265	0.474	0.015	0.261	0.482	−0.014	0.250
$X_{10}$	0.109	0.548	0.115	−0.010	0.490	0.250	−0.012	0.493	0.258	0.005	0.492	0.258
$X_{11}$	−0.149	−0.085	0.511	−0.472	−0.004	0.267	−0.473	0	0.271	−0.465	0.002	0.282
$X_{12}$	0.217	−0.403	0.260	−0.008	−0.493	0.238	−0.009	−0.490	0.230	0.003	−0.492	0.231
Factor correlations												
	Oblimin			Target original			Target			Semi-specified target		
	$\eta_{1}$	$\eta_{2}$	ARS	$\eta_{1}$	$\eta_{2}$	ARS	$\eta_{1}$	$\eta_{2}$	ARS	$\eta_{1}$	$\eta_{2}$	ARS
$\eta_{1}$	1	0.072	−0.060	1	−0.003	−0.010	1	0	0.004	1	−0.001	0.001
$\eta_{2}$	0.072	1	0.063	−0.003	1	0.022	0	1	0.008	−0.001	1	0
ARS	−0.060	0.063	1	−0.010	0.022	1	0.004	0.008	1	0.001	0	1

*Note.* The “Target Original” loadings are the data-generating loadings, and, except for oblimin, the rotated loadings (below) are obtained by rotating toward the target specified in the corresponding columns of the top part of the table. ARS = acquiescence response style.

#### Uninformed Rotation

Uninformed simple structure rotation tries to achieve simple structure by minimizing a rotation criterion, without applying any user-specified expectations regarding the MM. Several oblique rotation criteria are available. One is (Direct) oblimin (Clarkson & Jennrich, 1988), which is widely used and offered by popular statistical packages (e.g., SPSS, STATA); others are promax (Hendrickson & White, 1964), promin (Lorenzo-Seva, 1999), and geomin (the default in Mplus; Asparouhov & Muthén, 2009; Yates, 1988).

In the example, we rotated the estimated unrotated loadings using oblimin,^3^ and the results are displayed in the bottom part of Table 1. The oblimin rotated loadings illustrate how, by using uninformed simple structure rotation, the original factor structure is not recovered. For example, items 4 and 8 load moderately on all factors, and, without further investigations, one might decide to erroneously discard these two items from the scale. This result is not suprising, as previous research already established that, in the case of items loading on multiple factors (here due to the ARS factor), uninformed simple structure rotation criteria perform sub-optimally (Ferrando & Seva, 2000; Lorenzo-Seva, 1999; Schmitt & Sass, 2011). It is interesting to observe how, in order to pursue simple structure, the rotation tries to separate the positive and negative poles of the two content factors.^4^ However, with only three factors this cannot be achieved, and, as a result, it produces many small and moderate cross-loadings that seem to correspond with such a tendency to separate the different poles of each content factor. For example, the loadings of $\eta_{1}$ that are negative in the population (i.e., items 3 and 7) become primary loadings on the third factor, whereas the negative loadings on $\eta_{2}$ (i.e., items 4 and 8) become moderate loadings on all factors.

#### Informed Rotation

In informed rotation approaches (e.g., target rotation; Browne, 2001) assumptions regarding the MM are translated into a user-specified target loading matrix. The loadings are, then, rotated to approximate this target loading matrix. The target does not need to be fully specified (i.e., some elements may be unspecified). The specified elements can be zero or take on any value for the non-zero loadings, but, in many practical applications, it is recommended to specify only the zero loadings as precise values for the non-zero loadings are rarely, if ever, known prior to estimating the model (Browne, 2001). Furthermore, some studies have highlighted the robustness of partially (or semi-) specified target rotation when the zero target values are left unspecified and the non-zero target values are misspecified (Myers et al., 2013, 2015), but the generalizability of these results to fully specified target rotation as well as to misspecification of the zero loadings (e.g., erroneously specifying a non-zero loading as zero) remains unclear (Garcia-Garzon et al., 2019). Note that, despite the exploratory evaluation of the scale’s MM, researchers often have at least some expectations on what the scale is measuring. In fact, the scale is developed to measure one or more content factors and the questions are specifically selected and attuned to do so. However, if researchers do not have such expectations, simplimax (Kiers, 1994) may be used to obtain an optimal, empirically derived semi-specified target for a given loading matrix, as well as the target-rotated loadings.

In the top part of Table 1, two different fully specified target matrices are displayed, that is, one with the data-generating values, and one in which the structure was specified using zeros and ones (as is often done in practice), and the corresponding rotated loadings are shown below these target matrices. In both cases, the rotated factor loadings as well as the factor correlations are well recovered, which highlights the suitability of informed rotation approaches in the presence of violations of simple structure, for instance, due to an ARS factor. In order to avoid misspecification of the unknown elements in the target, semi-specified target rotation can be used. Table 1 displays a semi-specified target matrix, specifying only the zero loadings, and the corresponding rotated loadings at the top and bottom part, respectively. The semi-specified target rotated loadings clearly show how zero and non-zero loadings as well as the factor correlations can be accurately recovered by specifying only part of the assumed factor structure in the target. Note that the loadings are recovered as well as with the rotation toward the fully specified target matrices.

### Potential Effects of ARS on Dimensionality Assessment

Until now, it was assumed that the additional ARS factor is retained, which might not always be the case in empirical applications. In fact, in EFA, the number of factors needs to be determined, and this decision generally relies on both “objective” criteria and subjective judgment (i.e., interpretability). A popular objective criterion for maximum likelihood (ML) factor analysis is the Bayesian Information Criterion (BIC; Schwarz, 1978), which is a function of how well a model fits the data (i.e., log-likelihood) and the model’s complexity (i.e., number of freely estimated parameters). For a model M , the BIC is calculated as

(5) BIC=−2LogLikelihood(M)+fpln(N)

where fp indicates the number of free (or estimated) parameters. Even though this criterion is commonly used in empirical practice to determine the number of factors, it may malfunction if multivariate normality cannot be safely assumed like in the case of ordered-categorical data, and in such cases, other approaches might be preferred. One of these alternative approaches is parallel analysis (PA; Horn, 1965), which takes sampling variability into account when selecting the number of factors. In PA, the eigenvalues of the factors estimated from an empirical (polychoric) correlation matrix are compared with the distribution of the eigenvalues estimated from a number of randomly generated (polychoric) correlation matrices (e.g., 20) of the same size as the empirical ones. Afterwards, a factor is retained if its eigenvalue is larger than a given cut-off in the distribution of the eigenvalues obtained from the randomly generated data. Another flexible procedure to determine the numbers of factors is the CHull procedure (Ceulemans & Kiers, 2006; Lorenzo-Seva et al., 2011), which can be considered as a generalization of the scree test (Cattell, 1966) that aims to balance model fit and complexity. This goal is achieved by first creating a plot of a goodness-of-fit measure against the degree of freedom and, then, selecting the solution which is on or close to the elbow of the higher boundary (convex hull) of the plot by means of a scree test. Lorenzo-Seva et al. (2011) suggested to use the *common part accounted for* index (CAF; l Lorenzo-Seva et al., 2011) as a goodness-of-fit measure. The CAF index expresses the degree to which the extracted factor(s) capture the common variance in the data. To calculate the CAF, first the Kaiser-Meyer-Olkin (KMO; Kaiser, 1970; Kaiser & Rice, 1974) index is calculated on the estimated residual correlation matrix $\Psi_{q}$ of a factor model with q factors. Then, the CAF for a model with q factors is obtained as $\mathrm{CA}F_{q}$ = 1–KMO $\left(\Psi_{q}\right)$ . The values of the CAF index range from 0 to 1, where values close to 1 indicate that no substantial amount of common variance is left in the residual matrix after extracting q factors. A crucial advantage of the CAF compared with other goodness-of-fit measures is that it can be calculated for a model with no factors, in which case the residual correlation matrix is equal to the empirical correlation matrix. For a detailed overview of “objective” model selection criteria, we refer the reader to Lorenzo-Seva et al. (2011).

Different aspects might play a role in retaining (i.e., selecting) an ARS as an additional factor. For example, various studies suggest that an ARS factor can be conceptualized as a weak factor (i.e., with items showing weak to moderate loadings; Danner et al., 2015; Ferrando et al., 2004), potentially making it harder to capture by “objective” model selection criteria. Furthermore, scales that are unbalanced (i.e., with only positively worded items) or partially balanced (i.e., with a few negatively worded items) might hamper the detection of an additional ARS factor as it would either be more difficult to differentiate it from the content factor(s), or even impossible in the case of unbalanced unidimensional scales (Ferrando & Lorenzo-Seva, 2010; Savalei & Falk, 2014).

**Figure 2.**
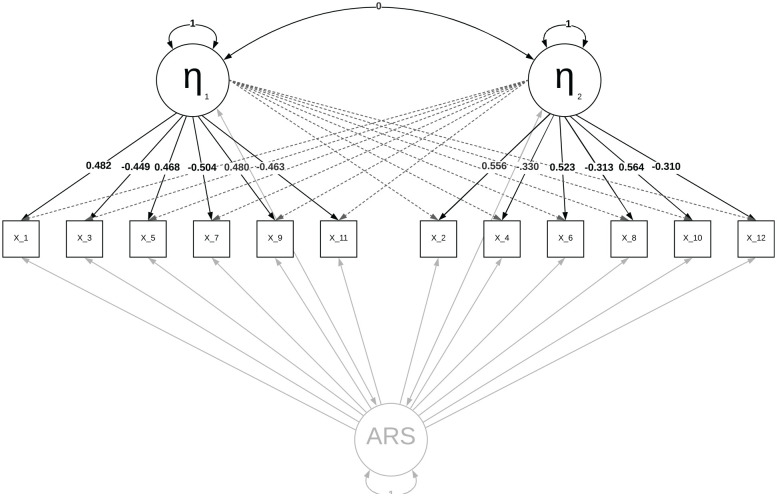
A Multidimensional Factor Model in Which the ARS Factor Is Ignored *Note.* The dotted lines indicate the zero loadings, the elements in gray were not included in the estimation, and the residuals are omitted for visual clarity. ARS = acquiescence response style.

Equally important, an ARS might seriously affect the assessment of the MM regardless of it being retained (i.e., an additional factor selected) in the model selection step or not. In fact, as shown in the illustrative example in section “Potential Effects Of ARS on Factor Rotation,” conclusions with regard to the MM are misleading if the ARS factor is retained and the loadings are rotated using uninformed simple structure rotation approaches. Alternatively, failure to select the ARS factor could result in biased loadings on the content factor(s) and bias in the factor correlations. An example of the latter is presented in Figure 2, where, after generating data using the model in Figure 1, a two-factor model was estimated (i.e., ignoring the ARS factor) and the estimated loadings were rotated using oblimin. The results displayed in Figure 2 indicate that not taking the ARS factor into account caused most loadings to be under/overestimated.

## Simulation Study

To evaluate the impact of an ARS on the assessment of the psychometric properties of unidimensional and multidimensional scales using EFA, a simulation study was conducted, where we assessed (a) the selected number of number of factors, and the recovery of factor loadings and correlations when ARS was (b) taken into count (i.e., extracted), and (c) ignored (i.e., not extracted). As a point of comparison a null scenario (i.e., without an ARS factor) was simulated, the results of which are reported in the Appendix.

The following six factors were manipulated:

- The number of subjects N at two levels: 250, 500.
- The number of categories C for each item at three levels: 3, 5, 7.
- The type of scale at two levels: balanced, unbalanced.
- The number of content factors Q at two levels: 1, 2.
- The number of items J per factor at two levels: 12, 24.
- The strength of the ARS factor at three levels: small, medium and large.

The sample size of 250 is in line with the recommended minimal sample for obtaining precise factor loading estimates with moderate item communalities (Fabrigar et al., 1999; MacCallum et al., 1999). Furthermore, the manipulated levels for the number of categories were chosen to represent the following: (a) items that can be treated as ordinal (i.e., three categories), (b) continuous (i.e., seven categories), or (c) both (i.e., five categories; Rhemtulla et al., 2012). In addition, both balanced and unbalanced scales were included, as the former are generally suggested and preferred to detect ARS (Ferrando & Lorenzo-Seva, 2010; Van Vaerenbergh & Thomas, 2013), whereas the latter is representative of most empirical applications (Ferrando & Lorenzo-Seva, 2010). Finally, both unidimensional and multidimensional scales were simulated. A full-factorial design was used with 2 (number of subjects) × 3 (number of categories) × 2 (type of scale) × 2 (number of factors) × 2 (number of items) × 3 (strength of ARS) = 144 conditions. For each condition, 100 replications were generated resulting in 14,400 data sets.

### Methods

#### Data Generation

We used a *Q*-dimensional normal ogive graded response model (noGRM) as the data-generating model to be able to use the mirt package (Chalmers, 2012) to generate the data, which allowed us to more flexibly generate data with varying numbers of categories while not substantially deviating from a factor model. In fact, parameters in the noGRM are directly related to those of a categorical factor model (Kamata & Bauer, 2008; Takane & De Leeuw, 1987). The population values of the model parameters reparametrized in a categorical confirmatory factor analysis fashion are displayed in Table 2. Note that the factors were not correlated in the data-generating model.

**Table 2.** Population Values Used in the Simulation Study.

Loadings				Thresholds											
	One factor	Two factors		Three categories		Five categories				Seven categories					
Item	λ	$\lambda_{C1}$	$\lambda_{C2}$	$\tau_{1}$	$\tau_{2}$	$\tau_{1}$	$\tau_{2}$	$\tau_{3}$	$\tau_{4}$	$\tau_{1}$	$\tau_{2}$	$\tau_{3}$	$\tau_{4}$	$\tau_{5}$	$\tau_{6}$
$X_{1}$	0.506	0.506	0	0	−2.000	0.875	−0.375	−1.625	−2.875	2.125	0.875	−0.375	−1.625	−2.875	−4.125
$X_{2}$	0.506	0	0.506	0.182	−1.818	1.057	−0.193	−1.443	−2.693	2.307	1.057	−0.193	−1.443	−2.693	−3.943
$X_{3}$	0.506	(–)0.506	0	0.364	−1.636	1.239	−0.011	−1.261	−2.511	2.489	1.239	−0.011	−1.261	−2.511	−3.761
$X_{4}$	0.506	0	(–)0.506	0.545	−1.455	1.420	0.170	−1.080	−2.330	2.670	1.420	0.170	−1.080	−2.330	−3.580
$X_{5}$	0.506	0.506	0	0.727	−1.273	1.602	0.352	−0.898	−2.148	2.852	1.602	0.352	−0.898	−2.148	−3.398
$X_{6}$	0.506	0	0.506	0.909	−1.091	1.784	0.534	−0.716	−1.966	3.034	1.784	0.534	−0.716	−1.966	−3.216
$X_{7}$	(–)0.506	(–)0.506	0	1.091	−0.909	1.966	0.716	−0.534	−1.784	3.216	1.966	0.716	−0.534	−1.784	−3.034
$X_{8}$	(–)0.506	0	(–)0.506	1.273	−0.727	2.148	0.898	−0.352	−1.602	3.398	2.148	0.898	−0.352	−1.602	−2.852
$X_{9}$	(–)0.506	0.506	0	1.455	−0.545	2.330	1.080	−0.170	−1.420	3.580	2.330	1.080	−0.170	−1.420	−2.670
$X_{10}$	(–)0.506	0	0.506	1.636	−0.364	2.511	1.261	0.011	−1.239	3.761	2.511	1.261	0.011	−1.239	−2.489
$X_{11}$	(–)0.506	(–)0.506	0	1.818	−0.182	2.693	1.443	0.193	−1.057	3.943	2.693	1.443	0.193	−1.057	−2.307
$X_{12}$	(–)0.506	0	(–)0.506	2.000	0	2.875	1.625	0.375	−0.875	4.125	2.875	1.625	0.375	−0.875	−2.125

To simulate balanced scales, for the content factor(s), half of the loadings were positive (i.e., indicative items), and the other half were negative (i.e., contra-indicative items), whereas all loadings were positive to simulate unbalanced scales. Furthermore, as displayed in Table 2, the distance between the first threshold of the easiest and the most difficult item was two *SDs* (e.g., for items with three categories, first threshold of item 1 = 0, and first threshold of item 12 = 2). To avoid estimation issues (e.g., non-convergence), we only accepted data sets where each item’s category contains at least a single observation. In the rare cases where a category was not present among the generated scores for a specific item, the entire data generation process was repeated until all response categories were observed.

**Figure 3.**
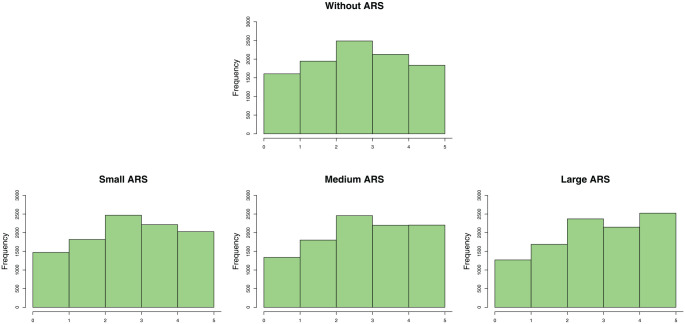
The Effects of the ARS Manipulations on a Five Categories Item With $\tau_{j}\;=\;\left\{-3.091,\;-1.091,\;-0.909,\;-2.909\right\}$ *Note.* ARS = acquiescence response style.

The ARS factor scores were sampled from a right-censored normal distribution. This distribution allowed us to simulate subjects who either did or did not show an ARS (i.e., have a positive or zero factor score on the ARS dimension), without allowing for scores representing a disagreeing tendency. Furthermore, with regard to the three levels of the ARS factor, the values of the loadings for the small, medium, and large ARS scenarios were 0.218, 0.343, and 0.506, respectively.^5^ The effects of a small, medium, and large ARS on the items’ univariate distribution are illustrated by the example shown in Figure 3, where data were generated for an item with five categories, 10,000 observations, and using the thresholds of the seventh item in Table 2. Clearly, the higher categories (i.e., 4 and 5) are more often selected as the strength of the ARS increases.

#### Data Analysis

The analyses proceeded as follows: first, for each generated data set, we estimated EFA models with up to three factors, in the case of unidimensional scales, and up to four factors, in the case of multidimensional scales. Furthermore, to study the effects of ARS when treating the data as ordinal or continuous (e.g., ordinal for three categories or approximately continuous for seven categories), the EFA models were estimated both for Pearson correlations and polychoric correlations. Note that the initial factor solutions were estimated with orthogonal factors and using maximum likelihood estimation.

Afterwards, three model selection criteria were considered to evaluate the number of dimensions (i.e., select among the three/four factor models), namely BIC, Parallel Analysis (PA), and the CHull using the CAF index as a goodness-of-fit measure (see section “Potential effects of ARS on dimensionality assessment”).^6^ For PA, we retained a factor if its eigenvalue was larger than a given $95^{\mathrm{th}}$ percentile in the distribution of eigenvalues obtained from 20 randomly generated data matrices. Specifically, we used the $95^{\mathrm{th}}$ percentile as the selected cut-off, as it is commonly used in practice (Lorenzo-Seva et al., 2011).

Next, irrespective of the results of the model selection procedures, the loadings for the models with and without the ARS factor were rotated using uninformed rotation approaches and informed rotation approaches. For uninformed rotation, we chose oblimin as it is a popular rotation approach available in most statistical software,^7^ and it allowed us to assess the effect of naively rotating toward simple structure when extracting an additional factor (i.e., as one would do when unaware of ARS).^8^ To avoid local optima, we performed oblimin rotation using the gradient projection algorithm with 10 random starts and delta = 0. For informed rotations, we used fully specified target (FST) and semi-specified target (SST) rotations, and the target matrices are displayed in Table 3. For FST rotation, the elements of both the content and the ARS factor were fully specified in the target matrices using ones and zeros for the non-zero and zero loadings, respectively, whereas only the zero loadings on the content factor were specified for SST. Also, FST and SST were used both when ARS factor was retained or not for multidimensional scales, whereas only FST was used when the ARS factor was retained for unidimensional scales. Oblique Procrustes rotation was used for each target rotation.

**Table 3.** Target Matrices.

Target Matrices								
	Unidimensional scales		Multidimensional scales					
	FST		FST			SST		
	$\eta_{1}$	ARS	$\eta_{1}$	$\eta_{2}$	ARS	$\eta_{1}$	$\eta_{2}$	ARS
$X_{1}$	1	1	1	0	1	NA	0	NA
$X_{2}$	1	1	0	1	1	0	NA	NA
$X_{3}$	1	1	(–)1	0	1	NA	0	NA
$X_{4}$	1	1	0	(–)1	1	0	NA	NA
$X_{5}$	1	1	1	0	1	NA	0	NA
$X_{6}$	1	1	0	1	1	0	NA	NA
$X_{7}$	(–)1	1	(–)1	0	1	NA	0	NA
$X_{8}$	(–)1	1	0	(–)1	1	0	NA	NA
$X_{9}$	(–)1	1	1	0	1	NA	0	NA
$X_{10}$	(–)1	1	0	1	1	0	NA	NA
$X_{11}$	(–)1	1	(–)1	0	1	NA	0	NA
$X_{12}$	(–)1	1	0	(–)1	1	0	NA	NA

*Note.* FST = Fully specified target; SST = Semi-specified target; ARS = acquiescence response style.

#### Outcome Measures

The true positive rate (TPR) was calculated for the BIC, PA, and CHull, both for the models estimated using polychoric correlations and Pearson correlations. Here, the TPR represents the proportion of selecting a two- or three-factor model for unidimensional and multidimensional scales, respectively—that is, the proportion of selecting the additional ARS factor.

Furthermore, the root mean square error (RMSE) between the estimated and true values of the factor loadings was calculated as $\mathrm{RMS}E_{\mathrm{loadings}}=\sqrt{\frac{1}{\mathrm{JQ}}\;\sum_{q=1}^{Q}\;\sum_{j=1}^{J}\;{\left({\hat{\lambda }}_{\mathrm{jq}}-\lambda_{\mathrm{jq}}\right)}^{2}}$ . Note that this was computed twice for each generated data set—that is, for the model excluding the ARS factor and the model including it (i.e., regardless of the number of factors suggested by the model selection criteria)—and both values were averaged across all replications in a cell of the factorial design.

Then, an RMSE was obtained for the content factor(s) ( $\mathrm{RMS}E_{\mathrm{loadingsC}}$ ) and the ARS factor^9^ ( $\mathrm{RMS}E_{\mathrm{loadingsARS}}$ ) when ARS was extracted, and only for the content factor(s) when ARS was not extracted. In addition, we evaluated whether ARS could cause items to load on more than one content factor simultaneously (i.e., cross-loadings), which would cause researchers to conclude that these items are not pure measurements of one factor. Therefore, for multidimensional scales, the recovery of the loadings that are zero in the data-generating model (i.e., on the content factors) was also assessed by calculating the mean maximum absolute bias (MMAB). Specifically, we first selected, for each rotation approach, the item with the maximum absolute difference between the estimated and the “true” (zero) loading, and then we averaged across data sets. In addition, the recovery of the factor correlations between content factors was calculated as $\mathrm{RMS}E_{\mathrm{FactorCorr}}=\sqrt{{\left({\hat{\phi }}_{\eta_{1}\eta_{2}}\;-\;\phi_{\eta_{1}\eta_{2}}\right)}^{2}}$ . Similarly to the factor loadings, this measure was computed twice for each generated data set in the conditions with multidimensional scales (i.e., for the model excluding the ARS factor and for the model including it), and averaged across all data sets in a cell of the factorial design.

The results for the model selection and recovery of factor loadings and factor correlations when ARS was not simulated (i.e., null scenario), are reported in the Appendix (Tables A1 – A4) as the performance of the model selection and rotation approaches in these conditions only serves as a comparison. In short, their performance was generally satisfactory in all conditions, with a TPR—in this case, equal to the proportion of selecting the correct number of content factors—of at least 0.90 for all model selection criteria, and $\mathrm{RMS}E_{\mathrm{loadingsC}}$ and $\mathrm{RMS}E_{\mathrm{FactorCorr}}$ < 0.1 for all rotation approaches.

#### Data Simulation, Software and Packages

The data were simulated and analyzed using R (R Core Team, 2013). Specifically, for generating the data, the R package mirt was used (Chalmers, 2012), while EFA and PA were conducted using the psych package (Revelle & Revelle, 2015). The CHull procedure was performed using the multichull package (Vervloet et al., 2017). For target rotation, we used a function based on Jennrich (2002), which, unlike the one in the popular R package psych , does not rescale the factors to improve agreement to the target. In fact, rescaling the factors would undesirably distort the FST-rotated loadings, that is, both zero and non-zero loadings are rescaled, and thus, increased to achieve agreement with the potentially misspecified values for the non-zero loadings.

### Results

#### Dimensionality Assessment

The TPR results for the different model selection criteria in the small, medium, and large ARS conditions largely overlapped between unidimensional and multidimensional scales. Hence, we only report the multidimensional scale results in Table 4 and Table 5 for balanced and unbalanced scales, respectively.^10^ Overall, the performance of the model selection criteria was mostly affected by the type of scale (i.e., balanced and unbalanced) and the strength of the ARS. The ARS factor was almost never retained in the conditions with unbalanced scales as indicated by the close-to-zero TPRs. These results align with and generalize those from Ferrando and Lorenzo-Seva (2010), who indicated that, for unidimensional unbalanced scales, the fit of a unidimensional model (i.e., without the additional ARS factor) would generally be acceptable as the content factor loadings absorb the ARS factor loadings. Our results extend this finding to multidimensional unbalanced scales, where the cross-loadings on content factors absorb the additional ARS factor, making the latter undetectable in the model selection step. For balanced scales, the additional ARS factor was mostly selected in the conditions with medium and large ARS, where both Pearson-based PA and CHull were equally sensitive or more sensitive than the BIC to this additional factor.^11^ However, polychoric-based PA rarely suggested to retain the additional ARS factor in the low- and medium-ARS conditions, which is in line with previous research that showed that polychoric-based PA generally underestimates the number of dimensions (Cho et al., 2009).

**Table 4.** Main Effects on Model Selection TPR for Multidimensional Balanced Scales in Function of Strength of the ARS and the Simulated Conditions.

Model selection multidimensional balanced scales																		
	Small ARS						Medium ARS						Large ARS					
	Pearson			Polychoric			Pearson			Polychoric			Pearson			Polychoric		
	CHull	BIC	PA	CHull	BIC	PA	CHull	BIC	PA	CHull	BIC	PA	CHull	BIC	PA	CHull	BIC	PA
*N* = 250	0.057	0	0.030	0.038	0	0	0.488	0.002	0.718	0.488	0.172	0.177	0.995	0.907	0.998	0.992	0.990	0.997
*N* = 500	0.045	0	0.058	0.047	0	0	0.788	0.350	0.957	0.743	0.717	0.078	1	0.998	1	1	1	0.993
C = 3	0.048	0	0.022	0.035	0	0	0.620	0.057	0.818	0.545	0.592	0.208	0.998	0.938	1	0.995	1	1
C = 5	0.052	0	0.035	0.050	0	0	0.610	0.200	0.812	0.612	0.350	0.105	0.995	0.920	0.998	0.992	0.985	0.988
C = 7	0.052	0	0.075	0.042	0	0	0.685	0.270	0.882	0.690	0.390	0.070	1	1	1	1	1	0.998
J = 12	0.065	0	0.042	0.050	0	0	0.517	0.067	0.723	0.488	0.280	0.083	0.995	0.905	0.998	0.992	0.990	0.990
J = 24	0.037	0	0.047	0.035	0	0	0.760	0.285	0.952	0.743	0.608	0.172	1	1	1	1	1	1

*Note.* TPR = true positive rate; ARS = acquiescence response style; CHull = convex hull based on the common part accounted for (CAF) index; BIC = Bayesian information criterion; PA = parallel analysis.

**Table 5.** Main Effects on Model Selection TPR for Multidimensional Unbalanced Scales in Function of Strength of the ARS and the Simulated Conditions.

Model selection multidimensional unbalanced scales																		
	Small ARS						Medium ARS						Large ARS					
	Pearson			Polychoric			Pearson			Polychoric			Pearson			Polychoric		
	CHull	BIC	PA	CHull	BIC	PA	CHull	BIC	PA	CHull	BIC	PA	CHull	BIC	PA	CHull	BIC	PA
*N* = 250	0.032	0	0.010	0.028	0	0	0.043	0	0.008	0.032	0.002	0	0.020	0	0.003	0.017	0.003	0
*N* = 500	0.017	0	0.007	0.013	0.003	0	0.017	0	0.002	0.015	0	0	0.020	0	0	0.022	0	0
C = 3	0.022	0	0.018	0.028	0.005	0	0.035	0	0.005	0.022	0.002	0	0.022	0	0.002	0.028	0.005	0
C = 5	0.022	0	0	0.012	0	0	0.030	0	0.005	0.020	0	0	0.022	0	0.002	0.015	0	0
C = 7	0.028	0	0.008	0.022	0	0	0.025	0	0.005	0.028	0	0	0.015	0	0	0.015	0	0
J = 12	0.038	0	0.013	0.027	0.003	0	0.043	0	0.010	0.038	0.002	0	0.030	0	0.003	0.033	0.003	0
J = 24	0.010	0	0.003	0.015	0	0	0.017	0	0	0.008	0	0	0.010	0	0	0.005	0	0

*Note.* TPR = true positive rate; ARS = acquiescence response style; CHull = convex hull based on the common part accounted for (CAF) index; BIC = Bayesian information criterion; PA = parallel analysis.

#### Bias With the Additional ARS Dimension

##### Factor Loadings

The $\mathrm{RMS}E_{\mathrm{loadingsC}}$ results using balanced scales are displayed in Tables 6 and 7 for unidimensional and multidimensional scales, respectively.^12^ FST rotation, for unidimensional scales, and both FST and SST rotation, for multidimensional scales, outperformed oblimin and resulted in an $\mathrm{RMS}E_{\mathrm{loadingsC}}$ that was always < 0.1, and even lower for EFA based on polychoric correlations. Oblimin rotation often resulted in highly biased loadings, especially in the case of unidimensional scales, where an $\mathrm{RMS}E_{\mathrm{loadingsC}}$ ≈ 0.2 was often observed. Note that this result is not particularly surprising as uninformed rotation approaches are known to perform sub-optimally when simple structure is violated (Ferrando & Seva, 2000; Lorenzo-Seva, 1999; Schmitt & Sass, 2011).

**Table 6.** $\mathrm{RMS}E_{\mathrm{loadingsC}}$ in Unidimensional Balanced Scales When the ARS Factor Is Extracted in Function of the Simulated Conditions.

Unidimensional balanced scales - $\mathrm{RMS}E_{\mathrm{loadingsC}}$ with ARS factor														
			Pearson						Polychoric					
			Small ARS		Medium ARS		Large ARS		Small ARS		Medium ARS		Large ARS	
*N*	J	C	Oblimin	FST	Oblimin	FST	Oblimin	FST	Oblimin	FST	Oblimin	FST	Oblimin	FST
**250**	**12**	**3**	0.223	0.035	0.221	0.036	0.212	0.032	0.202	0.024	0.198	0.026	0.191	0.032
		**5**	0.221	0.019	0.220	0.026	0.231	0.080	0.198	0.011	0.199	0.007	0.214	0.053
		**7**	0.215	0.023	0.212	0.020	0.212	0.051	0.222	0.011	0.215	0.011	0.204	0.040
	**24**	**3**	0.246	0.051	0.224	0.023	0.234	0.078	0.178	0.014	0.193	0.042	0.201	0.022
		**5**	0.241	0.028	0.218	0.016	0.228	0.063	0.213	0.007	0.210	0.018	0.210	0.037
		**7**	0.236	0.035	0.237	0.055	0.225	0.058	0.228	0.023	0.232	0.042	0.213	0.046
**500**	**12**	**3**	0.246	0.065	0.246	0.065	0.245	0.080	0.206	0.012	0.213	0.011	0.210	0.024
		**5**	0.239	0.024	0.252	0.056	0.220	0.036	0.225	0.007	0.226	0.030	0.202	0.010
		**7**	0.238	0.037	0.234	0.030	0.224	0.044	0.233	0.024	0.225	0.017	0.216	0.032
	**24**	**3**	0.242	0.050	0.250	0.067	0.233	0.051	0.210	0.011	0.225	0.015	0.198	0.011
		**5**	0.240	0.039	0.237	0.039	0.227	0.049	0.235	0.014	0.224	0.016	0.211	0.023
		**7**	0.251	0.042	0.239	0.041	0.219	0.026	0.245	0.030	0.232	0.029	0.208	0.014

*Note.* RMSE = root mean square error; ARS = acquiescence response style; FST = fully specified target. The bold entries were used to distinguish between design factors and do not refer to results.

**Table 7.** $\mathrm{RMS}E_{\mathrm{loadingsC}}$ in Multidimensional Balanced Scales When the ARS Factor Is Extracted in Function of the Simulated Conditions.

Multidimensional balanced scales— $\mathrm{RMS}E_{\mathrm{loadingsC}}$ with ARS factor Pearson polychoric																				
			Person									Polychoric								
			Small ARS			Medium ARS			Large ARS			Small ARS			Medium ARS			Large ARS		
*N*	J	C	Oblimin	FST	SST	Oblimin	FST	SST	Oblimin	FST	SST	Oblimin	FST	SST	Oblimin	FST	SST	Oblimin	FST	SST
**250**		**3**	0.080	0.016	0.052	0.092	0.021	0.043	0.114	0.034	0.040	0.042	0.041	0.015	0.058	0.032	0.010	0.083	0.014	0.010
	**12**	**5**	0.062	0.023	0.029	0.082	0.039	0.040	0.126	0.043	0.045	0.045	0.027	0.013	0.069	0.035	0.025	0.113	0.026	0.026
		**7**	0.063	0.015	0.033	0.078	0.033	0.024	0.121	0.026	0.023	0.056	0.019	0.025	0.070	0.031	0.016	0.119	0.020	0.014
		**3**	0.070	0.036	0.050	0.085	0.037	0.043	0.133	0.059	0.058	0.036	0.013	0.013	0.048	0.026	0.006	0.105	0.024	0.021
	**24**	**5**	0.052	0.023	0.035	0.064	0.021	0.026	0.139	0.046	0.044	0.034	0.014	0.018	0.047	0.012	0.008	0.132	0.029	0.025
		**7**	0.046	0.027	0.032	0.072	0.027	0.031	0.124	0.038	0.038	0.039	0.024	0.024	0.065	0.021	0.023	0.118	0.031	0.030
**500**		**3**	0.087	0.036	0.062	0.097	0.042	0.047	0.135	0.061	0.062	0.054	0.011	0.024	0.067	0.048	0.007	0.107	0.022	0.023
	**12**	**5**	0.052	0.026	0.026	0.081	0.033	0.041	0.108	0.035	0.034	0.036	0.023	0.009	0.063	0.019	0.022	0.096	0.018	0.014
		**7**	0.048	0.011	0.023	0.078	0.034	0.030	0.111	0.033	0.034	0.042	0.011	0.014	0.070	0.033	0.022	0.101	0.025	0.026
		**3**	0.056	0.039	0.044	0.092	0.050	0.053	0.124	0.045	0.044	0.019	0.013	0.006	0.059	0.021	0.014	0.098	0.016	0.006
	**24**	**5**	0.038	0.027	0.029	0.067	0.024	0.025	0.111	0.033	0.032	0.020	0.012	0.011	0.050	0.017	0.006	0.097	0.016	0.013
		**7**	0.037	0.026	0.029	0.070	0.029	0.032	0.119	0.027	0.027	0.030	0.018	0.021	0.063	0.021	0.024	0.115	0.019	0.019

*Note.* RMSE = root mean square error; ARS = acquiescence response style; FST = fully specified target; SST = semi-specified target. The bold entries were used to distinguish between design factors and do not refer to results.

For multidimensional balanced scales, Table 8 displays the MMAB results for the zero loadings when the ARS factor is extracted.^13^ The MMAB was below 0.2 for informed rotation approaches, but not for oblimin rotation, for which MMAB was often > 0.2 in conditions with medium ARS and always > 0.3 in conditions with large ARS, and thus is larger than the commonly used cut-off of 0.2 for “non-ignorable” cross-loadings (Stevens, 1992).

**Table 8.** Main Effects on MMAB for Zero Loadings in Multidimensional Balanced Scales When the ARS Factor Is Extracted in Function of the Simulated Conditions.

Multidimensional balanced scales—MMAB with ARS factor																		
	Small ARS						Medium ARS						Large ARS					
	Pearson			Polychoric			Pearson			Polychoric			Pearson			Polychoric		
	Oblimin	FST	SST	Oblimin	FST	SST	Oblimin	FST	SST	Oblimin	FST	SST	Oblimin	FST	SST	Oblimin	FST	SST
*N* = 250	0.182	0.150	0.121	0.192	0.158	0.131	0.225	0.169	0.121	0.243	0.188	0.131	0.372	0.146	0.117	0.403	0.148	0.126
*N* = 500	0.123	0.104	0.087	0.134	0.108	0.093	0.194	0.120	0.085	0.208	0.133	0.091	0.325	0.099	0.082	0.346	0.103	0.088
C = 3	0.160	0.131	0.109	0.179	0.143	0.122	0.215	0.142	0.105	0.242	0.175	0.119	0.336	0.132	0.104	0.381	0.137	0.118
C = 5	0.149	0.129	0.102	0.157	0.131	0.110	0.202	0.140	0.102	0.214	0.152	0.109	0.339	0.124	0.100	0.361	0.126	0.105
C = 7	0.149	0.122	0.101	0.153	0.125	0.104	0.213	0.152	0.102	0.220	0.155	0.105	0.371	0.111	0.096	0.382	0.114	0.098
J = 12	0.158	0.125	0.095	0.169	0.129	0.102	0.210	0.156	0.096	0.227	0.174	0.105	0.336	0.125	0.091	0.360	0.123	0.098
J = 24	0.147	0.130	0.113	0.158	0.137	0.122	0.209	0.133	0.109	0.224	0.147	0.117	0.361	0.120	0.108	0.389	0.128	0.117

*Note.* MMAB = mean maximum absolute bias; ARS = acquiescence response style; FST = fully-specified target; SST = semi-specified target.

##### Factor Correlations

The $\mathrm{RMS}E_{\mathrm{FactorCorr}}$ results for balanced scales are displayed in Table 9.^14^ The $\mathrm{RMS}E_{\mathrm{FactorCorr}}$ was < 0.1 for all rotation approaches in all simulated conditions, which indicates that extracting an additional ARS factor did not seem to impact the factor correlation regardless of the type of rotation.

**Table 9.** Main Effects on $\mathrm{RMS}E_{\mathrm{FactorCorr}}$ in Function of the Strength of the ARS and the Simulated Conditions When ARS Is Extracted in Balanced Scales.

$\mathrm{RMS}E_{\mathrm{FactorCorr}}$ with ARS factor																		
	Small ARS						Medium ARS						Large ARS					
	Pearson			Polychoric			Pearson			Polychoric			Pearson			Polychoric		
	Oblimin	FST	SST	Oblimin	FST	SST	Oblimin	FST	SST	Oblimin	FST	SST	Oblimin	FST	SST	Oblimin	FST	SST
*N* = 250	0.013	0.018	0.019	0.012	0.011	0.019	0.014	0.023	0.039	0.008	0.014	0.041	0.007	0.008	0.047	0.002	0.004	0.045
*N* = 500	0.016	0.018	0.019	0.012	0.006	0.020	0.007	0.007	0.020	0.013	0.004	0.019	0.005	0.004	0.032	0.003	0.001	0.030
C = 3	0.012	0.019	0.012	0.005	0.010	0.012	0.004	0.013	0.026	0.012	0.008	0.026	0.007	0.007	0.044	0.004	0.002	0.043
C = 5	0.009	0.026	0.028	0.008	0.008	0.029	0.012	0.020	0.032	0.012	0.012	0.034	0.004	0.008	0.040	0.002	0.004	0.039
C = 7	0.022	0.010	0.015	0.023	0.007	0.016	0.014	0.012	0.030	0.008	0.008	0.030	0.008	0.003	0.034	0.001	0.002	0.032
J = 12	0.012	0.015	0.022	0.014	0.009	0.022	0.014	0.021	0.038	0.009	0.014	0.040	0.006	0.005	0.040	0.003	0.003	0.037
J = 24	0.017	0.020	0.015	0.010	0.008	0.016	0.007	0.009	0.020	0.012	0.004	0.020	0.006	0.006	0.039	0.002	0.002	0.038

*Note.* RMSE = root mean square error; ARS = acquiescence response style; FST = fully specified target; SST = semi-specified target.

#### Bias Without the Additional ARS Dimension

##### Factor Loadings

The $\mathrm{RMS}E_{\mathrm{loadingsC}}$ results for unidimensional and multidimensional scales when the ARS factor was not retained are reported in Tables 10 to 12. The $\mathrm{RMS}E_{\mathrm{loadingsC}}$ was often <0.1 in all conditions and for both uninformed and informed rotation approaches, which suggests that ignoring (i.e., not extracting) the ARS factor did not strongly affect the recovery of factor loadings. Moreover, when comparing the rotation approaches in the conditions with multidimensional scales, FST and SST generally performed as well as or better than oblimin, and, again, the loadings were more accurately recovered when the EFA models were estimated using polychoric correlations.

**Table 10.** $\mathrm{RMS}E_{\mathrm{loadingsC}}$ in Unidimensional Scales When the ARS Factor Is not Extracted in Function of the Simulated Conditions.

Unidimensional scales – $\mathrm{RMS}E_{\mathrm{loadingsC}}$ without ARS factor														
			Balanced scales						Unbalanced scales					
			Small ARS		Medium ARS		Large ARS		Small ARS		Medium ARS		Large ARS	
*N*	J	C	Pearson	Polychoric	Pearson	Polychoric	Pearson	Polychoric	Pearson	Polychoric	Pearson	Polychoric	Pearson	Polychoric
250	12	3	0.043	0.015	0.043	0.018	0.042	0.025	0.045	0.019	0.063	0.008	0.034	0.037
		5	0.028	0.006	0.033	0.007	0.091	0.065	0.032	0.009	0.021	0.015	0.009	0.031
		7	0.030	0.018	0.026	0.016	0.069	0.066	0.016	0.006	0.023	0.010	0.021	0.035
	24	3	0.054	0.012	0.025	0.040	0.083	0.029	0.042	0.020	0.044	0.019	0.016	0.054
		5	0.031	0.007	0.018	0.016	0.068	0.042	0.043	0.017	0.030	0.004	0.025	0.057
		7	0.038	0.026	0.057	0.045	0.063	0.051	0.052	0.039	0.022	0.010	0.043	0.058
500	12	3	0.071	0.017	0.070	0.016	0.087	0.032	0.044	0.017	0.042	0.020	0.013	0.056
		5	0.029	0.006	0.060	0.035	0.048	0.021	0.026	0.005	0.010	0.036	0.016	0.046
		7	0.042	0.029	0.034	0.021	0.058	0.047	0.004	0.016	0.015	0.004	0.014	0.028
	24	3	0.051	0.009	0.070	0.019	0.054	0.010	0.035	0.026	0.024	0.041	0.017	0.053
		5	0.041	0.016	0.043	0.021	0.055	0.033	0.028	0.005	0.015	0.014	0.010	0.031
		7	0.044	0.031	0.043	0.031	0.032	0.022	0.032	0.019	0.003	0.013	0.014	0.028

*Note.* RMSE = root mean square error; ARS = acquiescence response style.

**Table 11.** $\mathrm{RMS}E_{\mathrm{loadingsC}}$ in Multidimensional Balanced Scales When the ARS Factor Is not Extracted in Function of the Simulated Conditions.

Multidimensional balanced scales— $\mathrm{RMS}E_{\mathrm{loadingsC}}$ without ARS factor																				
			Pearson									Polychoric								
			Small ARS			Medium ARS			Large ARS			Small ARS			Medium ARS			Large ARS		
*N*	J	C	Oblimin	FST	SST	Oblimin	FST	SST	Oblimin	FST	SST	Oblimin	FST	SST	Oblimin	FST	SST	Oblimin	FST	SST
250	12	3	0.036	0.036	0.037	0.043	0.041	0.045	0.064	0.054	0.073	0.014	0.015	0.015	0.011	0.011	0.020	0.037	0.037	0.056
		5	0.022	0.032	0.024	0.040	0.053	0.042	0.070	0.064	0.077	0.009	0.022	0.013	0.022	0.042	0.027	0.054	0.051	0.064
		7	0.025	0.025	0.025	0.025	0.039	0.029	0.071	0.083	0.083	0.020	0.020	0.020	0.017	0.034	0.024	0.069	0.084	0.079
	24	3	0.045	0.045	0.045	0.043	0.044	0.045	0.079	0.078	0.090	0.009	0.011	0.011	0.011	0.016	0.016	0.047	0.063	0.069
		5	0.029	0.031	0.030	0.026	0.026	0.027	0.073	0.080	0.084	0.012	0.017	0.014	0.010	0.009	0.010	0.065	0.081	0.082
		7	0.028	0.031	0.029	0.032	0.033	0.033	0.072	0.076	0.085	0.021	0.026	0.023	0.025	0.026	0.027	0.070	0.078	0.086
500	12	3	0.052	0.052	0.053	0.052	0.053	0.054	0.085	0.080	0.093	0.014	0.014	0.015	0.021	0.026	0.026	0.052	0.047	0.066
		5	0.020	0.032	0.021	0.043	0.043	0.044	0.056	0.058	0.063	0.003	0.021	0.008	0.025	0.026	0.027	0.045	0.050	0.056
		7	0.017	0.019	0.018	0.036	0.037	0.037	0.052	0.047	0.059	0.010	0.012	0.011	0.028	0.031	0.030	0.047	0.041	0.055
	24	3	0.042	0.044	0.042	0.054	0.055	0.054	0.057	0.062	0.062	0.004	0.012	0.006	0.016	0.021	0.018	0.037	0.048	0.047
		5	0.028	0.030	0.028	0.027	0.027	0.028	0.051	0.055	0.053	0.010	0.014	0.011	0.010	0.012	0.012	0.039	0.046	0.042
		7	0.028	0.029	0.028	0.034	0.034	0.034	0.055	0.060	0.070	0.020	0.021	0.021	0.026	0.026	0.026	0.057	0.065	0.076

*Note.* RMSE = root mean square error; ARS = acquiescence response style; FST = fully specified target; SST = semi-specified target.

**Table 12.** $\mathrm{RMS}E_{\mathrm{loadingsC}}$ in Multidimensional Unbalanced Scales When the ARS Factor Is not Extracted in Function of the Simulated Conditions.

Multidimensional unbalanced scales - $\mathrm{RMS}E_{\mathrm{loadingsC}}$ without ARS factor																				
			Pearson									Polychoric								
			Small ARS			Medium ARS			Large ARS			Small ARS			Medium ARS			Large ARS		
*N*	J	C	Oblimin	FST	SST	Oblimin	FST	SST	Oblimin	FST	SST	Oblimin	FST	SST	Oblimin	FST	SST	Oblimin	FST	SST
250	12	3	0.032	0.039	0.031	0.035	0.040	0.035	0.015	0.060	0.015	0.013	0.023	0.014	0.035	0.040	0.035	0.045	0.051	0.046
		5	0.011	0.020	0.011	0.011	0.044	0.011	0.014	0.051	0.014	0.014	0.019	0.014	0.011	0.044	0.011	0.033	0.045	0.034
		7	0.010	0.015	0.009	0.019	0.024	0.019	0.029	0.042	0.030	0.006	0.012	0.006	0.019	0.024	0.019	0.039	0.045	0.040
	24	3	0.028	0.029	0.028	0.024	0.025	0.024	0.018	0.063	0.018	0.017	0.017	0.016	0.024	0.025	0.024	0.047	0.051	0.048
		5	0.033	0.034	0.033	0.011	0.044	0.011	0.030	0.041	0.031	0.015	0.018	0.015	0.011	0.044	0.011	0.053	0.053	0.054
		7	0.035	0.035	0.035	0.012	0.020	0.012	0.028	0.048	0.028	0.027	0.027	0.027	0.012	0.020	0.012	0.038	0.047	0.039
500	12	3	0.037	0.038	0.037	0.027	0.049	0.027	0.016	0.057	0.015	0.007	0.010	0.007	0.027	0.049	0.027	0.032	0.040	0.033
		5	0.013	0.019	0.013	0.010	0.011	0.010	0.011	0.046	0.012	0.013	0.016	0.013	0.010	0.011	0.010	0.033	0.041	0.033
		7	0.004	0.014	0.004	0.010	0.022	0.010	0.018	0.037	0.019	0.011	0.016	0.011	0.010	0.022	0.010	0.029	0.038	0.029
	24	3	0.025	0.033	0.025	0.025	0.035	0.025	0.014	0.056	0.014	0.025	0.027	0.025	0.025	0.035	0.025	0.039	0.042	0.039
		5	0.016	0.020	0.016	0.014	0.037	0.014	0.007	0.021	0.007	0.007	0.012	0.007	0.014	0.037	0.014	0.024	0.027	0.024
		7	0.015	0.022	0.015	0.005	0.019	0.004	0.010	0.049	0.010	0.006	0.017	0.006	0.005	0.019	0.004	0.017	0.043	0.017

*Note.* RMSE = root mean square error; ARS = acquiescence response style; FST = fully specified target; SST = semi-specified target.

The MMAB results for the zero loadings in balanced and unbalanced scales are displayed in Tables 13 and 14. For all rotation approaches, the MMAB was >0.2 when large ARS was simulated in balanced scales, which is larger than this commonly used cut-off for “non-ignorable” cross-loadings (Stevens, 1992). In contrast, ignoring ARS did not increase the MMAB in the conditions with unbalanced scales as indicated by the MMAB always < 0.2. In fact, in comparison to Table A18 (i.e., when extracting the ARS factor), MMAB is now smaller (when using oblimin and FST) or equally small (when using SST).

**Table 13.** Main Effects on MMAB for Zero Loadings in Multidimensional Balanced Scales When the ARS Factor Is not Extracted in Function of the Simulated Conditions.

Multidimensional balanced scales—MMAB with ARS factor																		
	Small ARS						Medium ARS						Large ARS					
	Pearson			Polychoric			Pearson			Polychoric			Pearson			Polychoric		
	Oblimin	FST	SST	Oblimin	FST	SST	Oblimin	FST	SST	Oblimin	FST	SST	Oblimin	FST	SST	Oblimin	FST	SST
*N* = 250	0.136	0.148	0.134	0.146	0.155	0.144	0.140	0.156	0.137	0.150	0.164	0.148	0.227	0.275	0.214	0.252	0.309	0.237
*N* = 500	0.093	0.104	0.092	0.100	0.107	0.099	0.100	0.106	0.099	0.108	0.113	0.107	0.150	0.175	0.144	0.171	0.203	0.163
C = 3	0.118	0.128	0.117	0.133	0.140	0.132	0.122	0.132	0.120	0.140	0.147	0.137	0.189	0.223	0.179	0.223	0.271	0.210
C = 5	0.114	0.129	0.113	0.121	0.132	0.119	0.117	0.131	0.116	0.125	0.136	0.123	0.177	0.208	0.170	0.196	0.234	0.188
C = 7	0.111	0.120	0.110	0.115	0.122	0.114	0.119	0.130	0.118	0.123	0.133	0.122	0.201	0.242	0.190	0.216	0.262	0.202
J = 12	0.109	0.122	0.107	0.117	0.127	0.114	0.119	0.136	0.117	0.129	0.143	0.126	0.189	0.230	0.178	0.211	0.260	0.196
J = 24	0.120	0.129	0.120	0.129	0.136	0.129	0.120	0.126	0.120	0.129	0.134	0.128	0.188	0.220	0.181	0.212	0.251	0.204

*Note.* MMAB = mean maximum absolute bias; ARS = acquiescence response style; FST = fully-specified target; SST = semi-specified target.

**Table 14.** Main Effects on MMAB for Zero Loadings in Multidimensional Unbalanced Scales When the ARS Factor Is not Extracted in Function of the Simulated Conditions.

Multidimensional unbalanced scales—MMAB without ARS factor																		
	Small ARS						Medium ARS						Large ARS					
	Pearson			Polychoric			Pearson			Polychoric			Pearson			Polychoric		
	Oblimin	FST	SST	Oblimin	FST	SST	Oblimin	FST	SST	Oblimin	FST	SST	Oblimin	FST	SST	Oblimin	FST	SST
*N* = 250	0.131	0.143	0.130	0.141	0.149	0.139	0.134	0.154	0.132	0.143	0.156	0.141	0.132	0.170	0.129	0.142	0.156	0.139
*N* = 500	0.091	0.103	0.091	0.098	0.105	0.098	0.092	0.115	0.091	0.099	0.112	0.098	0.094	0.138	0.093	0.101	0.123	0.100
C = 3	0.114	0.128	0.113	0.129	0.136	0.128	0.115	0.137	0.114	0.131	0.142	0.130	0.119	0.176	0.117	0.135	0.150	0.133
C = 5	0.111	0.122	0.110	0.118	0.125	0.117	0.113	0.142	0.111	0.118	0.137	0.117	0.109	0.140	0.108	0.115	0.128	0.114
C = 7	0.108	0.119	0.107	0.111	0.120	0.111	0.111	0.124	0.110	0.113	0.124	0.112	0.111	0.147	0.109	0.114	0.140	0.112
J = 12	0.106	0.119	0.104	0.114	0.123	0.113	0.109	0.131	0.108	0.117	0.131	0.115	0.111	0.153	0.109	0.119	0.137	0.117
J = 24	0.116	0.127	0.116	0.125	0.131	0.124	0.116	0.138	0.116	0.124	0.138	0.124	0.115	0.156	0.114	0.123	0.141	0.122

*Note.* MMAB = mean maximum absolute bias; ARS = acquiescence response style; FST = fully specified target; SST = semi-specified target.

##### Factor Correlations

The $\mathrm{RMS}E_{\mathrm{FactorCorr}}$ results for both balanced and unbalanced scales are displayed in Table 15. The recovery of the factor correlations was generally satisfactory for all rotation approaches. Specifically, $\mathrm{RMS}E_{\mathrm{FactorCorr}}$ < 0.1 in most conditions, and both when using Pearson and polychoric correlations. This result indicates that ignoring (i.e., not extracting) the additional ARS factor did not affect the factor correlations much.

**Table 15.** Main Effects on $\mathrm{RMS}E_{\mathrm{FactorCorr}}$ in Function of the Strength of the ARS and the Simulated Conditions When ARS Is not Extracted.

$\mathrm{RMS}E_{\mathrm{FactorCorr}}$ without ARS factor																		
	Small ARS						Medium ARS						Large ARS					
	Pearson			Polychoric			Pearson			Polychoric			Pearson			Polychoric		
	Oblimin	FST	SST	Oblimin	FST	SST	Oblimin	FST	SST	Oblimin	FST	SST	Oblimin	FST	SST	Oblimin	FST	SST
*N* = 250	0.006	0.020	0.007	0.003	0.009	0.008	0.006	0.025	0.029	0.005	0.022	0.029	0.012	0.095	0.096	0.010	0.145	0.096
*N* = 500	0.003	0.017	0.016	0.004	0.008	0.017	0.005	0.017	0.019	0.009	0.014	0.021	0.007	0.035	0.050	0.008	0.070	0.046
C = 3	0.003	0.024	0.015	0.004	0.008	0.016	0.006	0.027	0.019	0.006	0.017	0.020	0.008	0.052	0.089	0.008	0.116	0.089
C = 5	0.006	0.021	0.016	0.003	0.010	0.017	0.005	0.023	0.036	0.006	0.026	0.037	0.006	0.064	0.044	0.008	0.105	0.040
C = 7	0.004	0.010	0.004	0.004	0.008	0.005	0.005	0.012	0.018	0.008	0.012	0.019	0.016	0.079	0.086	0.011	0.101	0.083
Balanced	0.004	0.020	0.004	0.003	0.007	0.004	0.004	0.019	0.004	0.004	0.015	0.005	0.005	0.078	0.008	0.005	0.072	0.009
Unbalanced	0.004	0.017	0.020	0.004	0.010	0.021	0.007	0.022	0.044	0.010	0.022	0.045	0.015	0.051	0.138	0.013	0.142	0.133
J = 12	0.004	0.017	0.010	0.004	0.008	0.011	0.007	0.027	0.021	0.006	0.021	0.023	0.010	0.064	0.044	0.010	0.100	0.041
J = 24	0.004	0.020	0.014	0.003	0.009	0.014	0.004	0.014	0.027	0.008	0.016	0.027	0.010	0.065	0.102	0.008	0.114	0.101

*Note.* RMSE = root mean square error; ARS = acquiescence response style; FST = fully specified target; SST = semi-specified target.

### Conclusions

The simulation study assessed the performance of EFA with regard to the number of suggested factors as well as the recovery of factor loadings and correlations in the presence of ARS both when retaining the ARS as an additional factor or not. The results indicated that, in terms of model selection, the type of scale as well as the strength of the ARS were particularly impactful on the suggested number of factors to retain. In fact, for both unidimensional and multidimensional scales, the additional ARS factor was almost never captured when unbalanced scales were simulated. In the conditions with balanced scales, the additional ARS factor was mostly selected when its strength was medium or large, especially by Pearson-based PA and to a lesser extent by the BIC and the CHull. Thus, in case of balanced scales, selecting an additional factor that may be an ARS factor is a realistic scenario one should be aware of.

In terms of factor rotation, when the ARS factor was extracted in balanced scales, the choice of how to rotate is important. In fact, rotating to simple structure (i.e., oblimin) resulted in biased loadings, and the maximal bias on the zero loadings was particularly large. The latter results are relevant for empirical practice, where trying to pursue simple structure in balanced scales with an additional (but unacknowledged) ARS factor might lead to (a) the exclusion of items that seem to measure multiple factors (i.e., with cross-loadings), or (b) under/overestimation how well the items measure a content factor (i.e., biased primary loading). In contrast, the factor loadings of balanced scales were accurately recovered when using informed rotation approaches (i.e., fully and semi-specified target rotation), which shows that it pays off to be aware of the fact that an additional factor may be an ARS factor. Taken together, these findings suggest that ARS is often extracted as an additional factor in balanced scales and that, for these scales, rotating toward (part of) the assumed MM (i.e., using informed rotation approaches) suffices to accurately assess the MM of these scales. Note that, for multidimensional scales, rotating toward a semi-specified target matrix, where only the zero loadings on the content factors were specified, allowed to recover the scales’ MM as accurately as when rotating toward a fully specified target matrix. However, ignoring the ARS factor in multidimensional balanced scales generally resulted in large cross-loadings (irrespective of the rotation), whereas not extracting an additional ARS factor did not affect the factor loading recovery in unbalanced scales. Hence, in empirical practice, researchers should be aware of the fact that not retaining an additional ARS factor might lead to erroneous conclusions on the psychometric properties of the questionnaire items in a balanced scale.

## Discussion

Assessing the psychometric properties of self-report scales is essential to obtain valid measurements of individuals’ latent psychological constructs (i.e., factors). This requires investigating the MM by determining the number of factors, their structure (i.e., which factor is measured by which item) and whether items are pure measurements of one factor. These psychometric properties are commonly assessed by EFA, where it is necessary to (a) evaluate the number of factors to retain, and (b) solve rotational freedom to enhance the interpretability of these retained factors. By means of a simulation study, we showed that these two aspects are affected by an ARS among the respondents, and that these effects on factor loadings and cross-loadings are more severe for balanced than for unbalanced scales. In what follows, we discuss the implications of these results for empirical practice for the two types of scales separately.

For balanced scales, especially large ARS often resulted in selecting an additional factor. For these scales, when retained, it is crucial to realize that this additional factor may be an ARS factor and to take this into account in the rotation step. In fact, we showed that naively rotating toward simple structure (i.e., assuming that each item measures only one factor) resulted in biased loadings as well as “non-ignorable” cross-loadings. The latter might drive researchers using balanced scales to draw erroneous conclusions when assessing whether items are non-ambiguous measures of a single factor, and whether they should be excluded from the scale (or replaced). This is avoided by using informed rotation approaches, where the additional ARS factor is taken into account by fully or partially specifying *a priori* assumptions or expectations regarding the MM in a target rotation matrix, and specifying the additional factor as a factor with high loadings for all items or leaving it unspecified. Furthermore, in multidimensional balanced scales, not extracting a large ARS factor often resulted in large cross-loadings, irrespective of the rotation. Thus, to properly assess the psychometric properties of a balanced scale, we not only recommend to use informed rotation if an additional factor is extracted but we even advise to extract the additional factor irrespective of whether the model selection criteria suggest to do so and compare this solution (upon informed rotation) to the one without this additional factor. Note that this result is also relevant to researchers that aim to use exploratory structural equation modeling (ESEM; Asparouhov & Muthén, 2009), where the number of factors is commonly assumed to be known *a priori*, and one, thus, likely disregards the potential presence of an ARS factor.

For unbalanced scales, the additional ARS factor was seldom selected in the model selection step. Our findings align with those analytically derived by Ferrando and Lorenzo-Seva (2010) for unidimensional scales and generalize them to multidimensional scales, where the cross-loadings on the factors allow for much flexibility so that the additional ARS factor is easily “absorbed” by the content ones, and thus hardly ever (or never) retained as an additional factor. Furthermore, not extracting ARS as an additional factor did not affect the factor loadings and correlation much, and, thus, when evaluating these psychometric properties, researchers can simply ignore the potential factor. Nevertheless, one should not conclude that ignoring an additional ARS factor in unbalanced scales is completely harmless. It is important to bear in mind that ARS might influence individual estimates with regard to the measured factors (i.e., factor scores), which, however, were not part of our investigation.

In summary, these findings indicate that it is crucial for researchers to beware of ARS and, for balanced scales, it is best to extract this as an additional factor and take its nature into account when rotating the factors. For the latter, our advise is to use semi-specified target rotation as it proved to perform well, and it avoids the potential influence of miss-specifying the size of the primary loadings—even though such an influence was not found in this article (Myers et al., 2013, 2015). When researchers do not have assumptions regarding the MM, an optimal semi-specified target for a given loading matrix—as well as the target-rotated loadings—can be obtained using simplimax (Kiers, 1994). However, note that the performance of simplimax when extracting an additional ARS factor has not been evaluated, and it would be interesting to do so in future research.

While providing useful insights on the effects of ARS on EFA, the generalizability of these results is subject to certain limitations. For instance, in this study, we only considered fully balanced or unbalanced scales but not semi-balanced scales. The latter are not uncommon in psychological research as, for some psychological constructs, contra-indicative items may be harder to formulate without facing the risk of measuring something else (Van Vaerenbergh & Thomas, 2013). Moreover, de la Fuente and Abad (2020) recently assessed the effects of ARS on both EFA and random intercept factor analysis (RIFA; Maydeu-Olivares & Coffman, 2006) with partially unbalanced scales, and showed that factor loadings were severely affected when using EFA (but not RIFA), especially when the size of the loadings differed strongly between indicative and contra-indicative items. However, whether the additional ARS factor was suggested in the model selection step was not investigated by them, and, in future research, it would certainly be interesting to investigate whether the ARS factor would be suggested in the model selection step. An additional limitation of our study is that the data were simulated under conditions where the MMs did not include cross-loadings among the content factors. However, this does not entirely correspond to empirical practice, where cross-loadings are frequently encountered (Li et al., 2020). Cross-loadings can have an important impact, not only on the number of factors to retain in EFA (Li et al., 2020) but also on the performance of uninformed rotation approaches (Ferrando & Seva, 2000; Lorenzo-Seva, 1999; Schmitt & Sass, 2011). Also, we only used oblimin as an uninformed rotation approach; however, future research may investigate the performance of uninformed rotation approaches that are suitable for the evaluation of MMs that do not adhere to simple structure, like promin rotation or promax-based tandem II (Beauducel & Kersting, 2020; Lorenzo-Seva, 1999). Finally, in this article, we specifically focused on oblique rotations (i.e., allowing content and ARS factors to be correlated), which is in line with recent theoretical and empirical studies addressing the relationships between personality traits and acquiescence (detailed reviews can be found in Weijters et al., 2010 and Ferrando et al., 2016). However, it should be noted that, for certain psychological traits, a relation with acquiescence may be irrelevant or absent (McCrae & Costa, 1983; Messick & Frederiksen, 1958). Therefore, for some unidimensional scales measuring these traits, orthogonal rotations (i.e., not allowing the content and ARS factor to be correlated) may be appropriate. Ferrando et al. (2016) developed a procedure to test the orthogonality assumption between a content factor and an ARS factor in unidimensional scales when a “good” set of items measuring acquiescence is available (e.g., a pool of items selected from a validated scale that measures acquiescence). However, as the authors had indicated, further research may be needed to (a) develop these marker items, which are not often available in practice and (b) extend this approach to multidimensional scales. In addition, oblique rotation may be appropriate even when the correlation between acquiescence and psychological traits is irrelevant. In fact, one may expect that, when using an informed rotation approach (e.g., target rotation) on factors that include an ARS factor, correlations among a content and ARS factor will be accurately recovered, and thus that a “true” zero correlation between ARS and content factors will likely result in an close-to-zero correlation after rotation. However, when using an uninformed rotation approach (e.g., oblimin), the different factors are not accurately disentangled, making this a less relevant issue. Recovery of the ARS factor (i.e., factor loadings and correlation) was not the goal of this investigation, but may be worth investigating in future research.

## Supplemental Material

sj-docx-1-ebm-10.1177_00131644221089857 – Supplemental material for Awareness Is Bliss: How Acquiescence Affects Exploratory Factor Analysis Click here for additional data file. Supplemental material, sj-docx-1-ebm-10.1177_00131644221089857 for Awareness Is Bliss: How Acquiescence Affects Exploratory Factor Analysis by E. Damiano D’Urso, Jesper Tijmstra, Jeroen K. Vermunt and Kim De Roover in Educational and Psychological Measurement

## Notes

1. This distribution might not be realistic for the acquiescence response style (ARS) factor $\eta_{i_{\mathrm{ARS}}}$ , if one keeps in mind that a score <0 would indicate a tendency to disagree. A more suitable distribution for ARS will be considered when generating the data in the “Simulation Study” section.

2. Note that the multidimensional factor model depicted in Figure 1 is substantively different from a bi-factor model (i.e., with a general factor; Holzinger & Swineford, 1937) due to the differences in sign for the loadings that are negative for the content factors and positive for the ARS factor, while for unbalanced scales (i.e., only positive loadings), the model in Figure 1 will be mathematically equivalent to a bi-factor model. As both types of scales (i.e., balanced and unbalanced) will be addressed in this article, bi-factor rotation approaches will not be discussed.

3. Note that, to avoid local optima, oblimin rotation was performed using the gradient projection algorithm with 10 random starts and delta = 0.

4. DiStefano and Motl (2006, 2009) already noted that results from analytical rotations, such as oblimin, may be confounded by a method effect when responses differ due to item wording (e.g., indicative or contra-indicative).

5. The loading values are converted from discrimination parameters of 0.38, 0.62, and 1, which were chosen such that the ARS factor affected the item responses drastically less than, less than or as much as (one of) the content factors, respectively. Note that, for a unidimensional noGRM, a discrimination parameter $\alpha_{j}$ can be converted to a factor loading $\lambda_{j}$ as $\lambda_{j}=\frac{\alpha_{j}}{\sqrt{1\;+\;\alpha_{j}^{2}}}$ (Kamata & Bauer, 2008).

6. Note that the *multichull* package imposes a minimal proportional increase in fit for a more complex model to be included in the hull (see Vervloet et al., 2017 for more details). By default, this minimal increase is set to 0.01. For the simulation study, we lowered it to 0.001, because this minimal value was not used in Lorenzo-Seva et al. (2011) and a value of 0.01 left the CHull insensitive to small ARS factors.

7. We preferred oblimin over geomin (Yates, 1988) due to geomin’s sensitivity to local minima (Asparouhov & Muthén, 2009; Browne, 2001). Note that oblimin performs at least as good as alternative rotation criteria when the measurement model underlying the data adhere to simple structure (Lorenzo-Seva, 2000).

8. For the purpose of evaluating the loadings recovery, the signs of the oblimin rotated factor loadings were reflected to match the ones used to generated the data.

9. Note that the variance of a right-censored normal distribution is smaller than the identification restrictions imposed on the variance of each factor (i.e., imposing them to equal 1). As a consequence, the loadings on the ARS factor seem to be underestimated. Therefore, in computing $\mathrm{RMS}E_{\mathrm{loadingsARS}}$ , the values of the estimated loadings on the ARS factor were compared with the values of the original loadings rescaled by the variance of a right-censored normal distribution. That is, we multiplied the value of the original loadings on the ARS factor by the *SD* of a right-censored normal distribution, which is ≈0.583. This resulted in loadings on the ARS factor of 0.128, 0.200, and 0.295 for the small, medium, and large ARS conditions, respectively.

10. The complete results, including those for unidimensional scales, can be found in the Appendix in Tables A5 to A11.

11. Note that, for the CHull, we visually inspected the cases where a solution could not be selected because the hull contained only two points. This happened in around 25% of the cases for the conditions with large ARS and balanced scales, and it was due to a slight decrease in the common part accounted for (CAF) index in the model with four factors in comparison to the three-factor model, which, thus, was not included in the hull. Visual inspection of these cases showed that the elbow was clearly visible for the model with three factors, and thus, we regarded these cases as having selected the correct number of factors.

12. Note that as the additional ARS factor was (almost) never selected for unbalanced scales we reported the $\mathrm{RMS}E_{\mathrm{loadingsC}}$ results for those conditions in the Appendix in Tables A12 to A13. In addition, we also reported the $\mathrm{RMS}E_{\mathrm{loadingsARS}}$ results for both balanced and unbalanced scales in the Appendix in Tables A14 to A17.

13. The results for unbalanced scales are displayed in Table A18 in the Appendix.

14. The $\mathrm{RMS}E_{\mathrm{FactorCorr}}$ for unbalanced scales are displayed in Table A19.
