# Supplementary material for: Awareness Is Bliss: How Acquiescence Affects Exploratory Factor Analysis
Source: Educ Psychol Meas. 2022 May 16;83(3):433–72. doi: 10.1177/00131644221089857 (PMC10177316; doi:10.1177/00131644221089857)
Supplement: sj-docx-1-ebm-10.1177_00131644221089857 – Supplemental material for Awareness Is Bliss: How Acquiescence Affects Exploratory Factor Analysis [file sj-docx-1-ebm-10.1177_00131644221089857.docx]

Agreeing response style and exploratory factor analysis

## 1 Appendix

Table 1: Model selection TPR for unidimensional scales in the null scenario in function of the simulated conditions

!

|  |  |  | **Model selection unidimensional scales in the null scenario** | | | | | | | | | | | |
| --- | --- | --- | --- | --- | --- | --- | --- | --- | --- | --- | --- | --- | --- | --- |
|  |  |  | **Balanced Scales** | | | | | | **Unbalanced scales** | | | | | |
|  |  |  | **Pearson** | | | **Polychoric** | | | **Pearson** | | | **Polychoric** | | |
| **N** | **J** | **C** | **CHull** | **BIC** | **PA** | **CHull** | **BIC** | **PA** | **CHull** | **BIC** | **PA** | **CHull** | **BIC** | **PA** |
| ***250** | 3***12** | **3** | 0.953 | 1 | 0.930 | 0.988 | 1 | 1 | 0.989 | 1 | 0.930 | 0.977 | 0.990 | 1 |
|  |  | **5** | 0.965 | 1 | 0.960 | 0.988 | 1 | 1 | 0.989 | 1 | 0.970 | 1 | 1 | 1 |
|  |  | **7** | 1 | 1 | 0.950 | 0.989 | 1 | 1 | 0.988 | 1 | 0.980 | 0.966 | 1 | 1 |
|  | 3***24** | **3** | 0.990 | 1 | 0.990 | 1 | 1 | 1 | 1 | 1 | 1 | 1 | 1 | 1 |
|  |  | **5** | 1 | 1 | 1 | 0.990 | 1 | 1 | 0.990 | 1 | 1 | 1 | 1 | 1 |
|  |  | **7** | 1 | 1 | 0.990 | 1 | 1 | 1 | 1 | 1 | 1 | 1 | 1 | 1 |
| ***500** | 3***12** | **3** | 1 | 1 | 0.900 | 1 | 1 | 1 | 0.978 | 1 | 0.960 | 0.976 | 1 | 1 |
|  |  | **5** | 1 | 1 | 0.950 | 0.989 | 1 | 1 | 1 | 1 | 0.980 | 1 | 1 | 1 |
|  |  | **7** | 0.989 | 1 | 0.950 | 0.977 | 1 | 1 | 1 | 1 | 0.960 | 0.989 | 1 | 1 |
|  | 3***24** | **3** | 1 | 1 | 0.990 | 1 | 1 | 1 | 1 | 1 | 0.990 | 1 | 1 | 1 |
|  |  | **5** | 1 | 1 | 0.990 | 1 | 1 | 1 | 1 | 1 | 1 | 1 | 1 | 1 |
|  |  | **7** | 1 | 1 | 1 | 1 | 1 | 1 | 1 | 1 | 1 | 1 | 1 | 1 |

[flushleft] Note. CHull = convex hull based on the Common Part Accounted For (CAF) index; BIC = Bayesian Information Criterion; PA = parallel analysis.

Table 2: $RMSE_{loadingsC}$ in unidimensional scales for the null scenario in function of the simulated conditions

!

|  |  |  | **$RMSE_{loadingsC}$ - Null scenario** | | | |
| --- | --- | --- | --- | --- | --- | --- |
|  |  |  | **Balanced scales** | | **Unbalanced scales** | |
| **N** | **J** | **C** | **Pearson** | **Polychoric** | **Pearson** | **Polychoric** |
| ***250** | 3***12** | **3** | 0.066 | 0.013 | 0.067 | 0.013 |
|  |  | **5** | 0.024 | 0.006 | 0.035 | 0.009 |
|  |  | **7** | 0.036 | 0.024 | 0.020 | 0.009 |
|  | 3***24** | **3** | 0.068 | 0.013 | 0.066 | 0.011 |
|  |  | **5** | 0.015 | 0.015 | 0.034 | 0.009 |
|  |  | **7** | 0.039 | 0.026 | 0.019 | 0.007 |
| ***500** | 3***12** | **3** | 0.040 | 0.019 | 0.055 | 0.006 |
|  |  | **5** | 0.013 | 0.016 | 0.013 | 0.016 |
|  |  | **7** | 0.037 | 0.024 | 0.003 | 0.013 |
|  | 3***24** | **3** | 0.046 | 0.014 | 0.055 | 0.005 |
|  |  | **5** | 0.030 | 0.005 | 0.025 | 0.004 |
|  |  | **7** | 0.046 | 0.033 | 0.022 | 0.010 |

Table 3: Model selection TPR for multidimensional scales in the null scenario in function of the simulated conditions

!

|  |  |  | **Model selection multidimensional scales in the null scenario** | | | | | | | | | | | |
| --- | --- | --- | --- | --- | --- | --- | --- | --- | --- | --- | --- | --- | --- | --- |
|  |  |  | **Balanced Scales** | | | | | | **Unbalanced scales** | | | | | |
|  |  |  | **Pearson** | | | **Polychoric** | | | **Pearson** | | | **Polychoric** | | |
| **N** | **J** | **C** | **CHull** | **BIC** | **PA** | **CHull** | **BIC** | **PA** | **CHull** | **BIC** | **PA** | **CHull** | **BIC** | **PA** |
| ***250** | 3***12** | **3** | 0.930 | 1 | 0.980 | 0.930 | 1 | 1 | 0.970 | 0.980 | 0.970 | 0.960 | 1 | 1 |
|  |  | **5** | 0.960 | 1 | 0.990 | 0.980 | 1 | 1 | 0.970 | 1 | 0.970 | 0.930 | 1 | 1 |
|  |  | **7** | 0.950 | 1 | 0.980 | 0.960 | 1 | 1 | 0.970 | 1 | 0.990 | 0.950 | 1 | 1 |
|  | 3***24** | **3** | 0.980 | 1 | 1 | 0.970 | 1 | 1 | 0.980 | 1 | 1 | 0.950 | 1 | 1 |
|  |  | **5** | 0.980 | 1 | 1 | 0.990 | 1 | 1 | 0.980 | 1 | 1 | 0.960 | 1 | 1 |
|  |  | **7** | 0.980 | 1 | 1 | 0.960 | 1 | 1 | 0.970 | 1 | 0.990 | 1 | 1 | 1 |
| ***500** | 3***12** | **3** | 0.960 | 1 | 0.990 | 0.960 | 1 | 1 | 0.980 | 1 | 0.980 | 0.990 | 1 | 1 |
|  |  | **5** | 0.960 | 1 | 0.990 | 0.950 | 1 | 1 | 0.970 | 1 | 0.980 | 1 | 1 | 1 |
|  |  | **7** | 0.960 | 1 | 1 | 0.970 | 1 | 1 | 1 | 1 | 0.990 | 0.940 | 1 | 1 |
|  | 3***24** | **3** | 1 | 1 | 0.990 | 0.990 | 1 | 1 | 1 | 1 | 1 | 0.990 | 1 | 1 |
|  |  | **5** | 0.990 | 1 | 1 | 0.990 | 1 | 1 | 1 | 1 | 1 | 0.990 | 1 | 1 |
|  |  | **7** | 1 | 1 | 1 | 0.970 | 1 | 1 | 1 | 1 | 1 | 1 | 1 | 1 |

[flushleft] Note. CHull = convex hull based on the Common Part Accounted For (CAF) index; BIC = Bayesian Information Criterion; PA = parallel analysis.

Table 4: $RMSE_{loadingsC}$ in multidimensional scales for the null scenario in function of the simulated conditions

!

|  |  |  | **Multidimensional scales - $RMSE_{loadings}$ in the Null scenario** | | | | | | | | | | | |
| --- | --- | --- | --- | --- | --- | --- | --- | --- | --- | --- | --- | --- | --- | --- |
|  |  |  | **Balanced scales** | | | | | | **Unbalanced scales** | | | | | |
|  |  |  | **Pearson** | | | **Polychoric** | | | **Pearson** | | | **Polychoric** | | |
| **N** | **J** | **C** | **Oblimin** | **FST** | **SST** | **Oblimin** | **FST** | **SST** | **Oblimin** | **FST** | **SST** | **Oblimin** | **FST** | **SST** |
| ***250** | 3***12** | **3** | 0.037 | 0.030 | 0.032 | 0.008 | 0.009 | 0.011 | 0.045 | 0.037 | 0.034 | 0.008 | 0.008 | 0.006 |
|  |  | **5** | 0.013 | 0.027 | 0.014 | 0.008 | 0.020 | 0.011 | 0.032 | 0.041 | 0.022 | 0.017 | 0.029 | 0.012 |
|  |  | **7** | 0.026 | 0.021 | 0.023 | 0.019 | 0.015 | 0.017 | 0.014 | 0.014 | 0.011 | 0.006 | 0.009 | 0.005 |
|  | 3***24** | **3** | 0.040 | 0.042 | 0.031 | 0.012 | 0.019 | 0.011 | 0.032 | 0.043 | 0.024 | 0.012 | 0.022 | 0.010 |
|  |  | **5** | 0.025 | 0.015 | 0.018 | 0.019 | 0.014 | 0.017 | 0.029 | 0.022 | 0.021 | 0.012 | 0.009 | 0.009 |
|  |  | **7** | 0.019 | 0.015 | 0.016 | 0.013 | 0.010 | 0.011 | 0.016 | 0.016 | 0.013 | 0.009 | 0.010 | 0.007 |
| ***500** | 3***12** | **3** | 0.035 | 0.026 | 0.027 | 0.009 | 0.007 | 0.009 | 0.039 | 0.030 | 0.027 | 0.015 | 0.013 | 0.011 |
|  |  | **5** | 0.011 | 0.008 | 0.010 | 0.011 | 0.009 | 0.011 | 0.020 | 0.017 | 0.015 | 0.007 | 0.008 | 0.005 |
|  |  | **7** | 0.011 | 0.020 | 0.011 | 0.005 | 0.014 | 0.005 | 0.005 | 0.007 | 0.004 | 0.011 | 0.010 | 0.008 |
|  | 3***24** | **3** | 0.035 | 0.038 | 0.027 | 0.008 | 0.014 | 0.008 | 0.041 | 0.031 | 0.030 | 0.007 | 0.007 | 0.006 |
|  |  | **5** | 0.016 | 0.023 | 0.014 | 0.006 | 0.012 | 0.006 | 0.019 | 0.018 | 0.015 | 0.004 | 0.007 | 0.004 |
|  |  | **7** | 0.022 | 0.022 | 0.017 | 0.014 | 0.015 | 0.011 | 0.012 | 0.010 | 0.009 | 0.005 | 0.004 | 0.003 |

Table 5: Main effects on model selection TPR for unidimensional scales in function of strength of the ARS and the simulated conditions

!

|  | **Model Selection Unidimensional Scales** | | | | | | | | | | | | | | | | | |
| --- | --- | --- | --- | --- | --- | --- | --- | --- | --- | --- | --- | --- | --- | --- | --- | --- | --- | --- |
|  | **Small ARS** | | | | | | **Medium ARS** | | | | | | **Large ARS** | | | | | |
|  | **Pearson** | | | **Polychoric** | | | **Pearson** | | | **Polychoric** | | | **Pearson** | | | **Polychoric** | | |
|  | **CHull** | **BIC** | **PA** | **CHull** | **BIC** | **PA** | **CHull** | **BIC** | **PA** | **CHull** | **BIC** | **PA** | **CHull** | **BIC** | **PA** | **CHull** | **BIC** | **PA** |
| **N = 250** | 0.014 | 0 | 0.038 | 0.012 | 0.001 | 0 | 0.084 | 0.005 | 0.318 | 0.092 | 0.039 | 0.041 | 0.440 | 0.476 | 0.502 | 0.420 | 0.496 | 0.498 |
| **N = 500** | 0.012 | 0 | 0.083 | 0.013 | 0 | 0 | 0.232 | 0.129 | 0.481 | 0.229 | 0.277 | 0.007 | 0.458 | 0.499 | 0.506 | 0.448 | 0.501 | 0.498 |
| **C = 3** | 0.009 | 0 | 0.041 | 0.010 | 0.001 | 0 | 0.081 | 0.004 | 0.380 | 0.099 | 0.191 | 0.015 | 0.418 | 0.471 | 0.509 | 0.385 | 0.496 | 0.496 |
| **C = 5** | 0.016 | 0 | 0.071 | 0.019 | 0 | 0 | 0.164 | 0.099 | 0.379 | 0.156 | 0.134 | 0.006 | 0.443 | 0.492 | 0.501 | 0.437 | 0.499 | 0.499 |
| **C = 7** | 0.015 | 0 | 0.069 | 0.009 | 0 | 0 | 0.230 | 0.099 | 0.440 | 0.227 | 0.149 | 0.050 | 0.487 | 0.499 | 0.501 | 0.480 | 0.500 | 0.500 |
| **Balanced** | 0.020 | 0 | 0.105 | 0.015 | 0.001 | 0 | 0.312 | 0.134 | 0.782 | 0.315 | 0.315 | 0.048 | 0.893 | 0.975 | 0.995 | 0.856 | 0.996 | 0.997 |
| **Unbalanced** | 0.006 | 0 | 0.016 | 0.010 | 0 | 0 | 0.005 | 0 | 0.017 | 0.007 | 0.001 | 0 | 0.005 | 0 | 0.012 | 0.012 | 0.001 | 0 |
| **J = 12** | 0.021 | 0 | 0.065 | 0.019 | 0.001 | 0 | 0.103 | 0.006 | 0.352 | 0.096 | 0.073 | 0.012 | 0.417 | 0.475 | 0.507 | 0.405 | 0.497 | 0.497 |
| **J = 24** | 0.006 | 0 | 0.056 | 0.006 | 0 | 0 | 0.214 | 0.128 | 0.447 | 0.225 | 0.243 | 0.036 | 0.481 | 0.500 | 0.501 | 0.463 | 0.500 | 0.500 |

[flushleft] Note. CHull = convex hull based on the Common Part Accounted For (CAF) index; BIC = Bayesian Information Criterion; PA = parallel analysis.

Table 6: Model selection TPR for unidimensional scales in function of small ARS and the simulated conditions

!

|  |  |  | **Model selection unidimensional scales with small ARS** | | | | | | | | | | | |
| --- | --- | --- | --- | --- | --- | --- | --- | --- | --- | --- | --- | --- | --- | --- |
|  |  |  | **Balanced Scales** | | | | | | **Unbalanced scales** | | | | | |
|  |  |  | **Pearson** | | | **Polychoric** | | | **Pearson** | | | **Polychoric** | | |
| **N** | **J** | **C** | **CHull** | **BIC** | **PA** | **CHull** | **BIC** | **PA** | **CHull** | **BIC** | **PA** | **CHull** | **BIC** | **PA** |
| ***250** | 3***12** | **3** | 0.022 | 0 | 0.040 | 0.011 | 0.010 | 0 | 0.022 | 0 | 0.040 | 0.034 | 0 | 0 |
|  |  | **5** | 0.032 | 0 | 0.100 | 0.022 | 0 | 0 | 0 | 0 | 0.030 | 0.024 | 0 | 0 |
|  |  | **7** | 0.033 | 0 | 0.030 | 0.011 | 0 | 0 | 0.011 | 0 | 0.030 | 0.011 | 0 | 0 |
|  | 3***24** | **3** | 0.010 | 0 | 0.050 | 0.010 | 0 | 0 | 0 | 0 | 0 | 0 | 0 | 0 |
|  |  | **5** | 0.010 | 0 | 0.050 | 0.010 | 0 | 0 | 0.010 | 0 | 0.020 | 0.010 | 0 | 0 |
|  |  | **7** | 0.020 | 0 | 0.060 | 0 | 0 | 0 | 0 | 0 | 0 | 0 | 0 | 0 |
| ***500** | 3***12** | **3** | 0 | 0 | 0.040 | 0 | 0 | 0 | 0.011 | 0 | 0.020 | 0.011 | 0 | 0 |
|  |  | **5** | 0.065 | 0 | 0.180 | 0.055 | 0 | 0 | 0.011 | 0 | 0.020 | 0.011 | 0 | 0 |
|  |  | **7** | 0.033 | 0 | 0.230 | 0.022 | 0 | 0 | 0.010 | 0 | 0.020 | 0.021 | 0 | 0 |
|  | 3***24** | **3** | 0.010 | 0 | 0.130 | 0.010 | 0 | 0 | 0 | 0 | 0.010 | 0 | 0 | 0 |
|  |  | **5** | 0 | 0 | 0.170 | 0.020 | 0 | 0 | 0 | 0 | 0 | 0 | 0 | 0 |
|  |  | **7** | 0.010 | 0 | 0.180 | 0.010 | 0 | 0 | 0 | 0 | 0 | 0 | 0 | 0 |

[flushleft] Note. CHull = convex hull based on the Common Part Accounted For (CAF) index; BIC = Bayesian Information Criterion; PA = parallel analysis.

Table 7: Model selection TPR for unidimensional scales in function of medium ARS and the simulated conditions

!

|  |  |  | **Model selection unidimensional scales with medium ARS** | | | | | | | | | | | |
| --- | --- | --- | --- | --- | --- | --- | --- | --- | --- | --- | --- | --- | --- | --- |
|  |  |  | **Balanced Scales** | | | | | | **Unbalanced scales** | | | | | |
|  |  |  | **Pearson** | | | **Polychoric** | | | **Pearson** | | | **Polychoric** | | |
| **N** | **J** | **C** | **CHull** | **BIC** | **PA** | **CHull** | **BIC** | **PA** | **CHull** | **BIC** | **PA** | **CHull** | **BIC** | **PA** |
| ***250** | 3***12** | **3** | 0.073 | 0 | 0.400 | 0.095 | 0.100 | 0.070 | 0.033 | 0 | 0.060 | 0.012 | 0.010 | 0 |
|  |  | **5** | 0.144 | 0 | 0.400 | 0.096 | 0 | 0.020 | 0 | 0 | 0.030 | 0.034 | 0 | 0 |
|  |  | **7** | 0.141 | 0 | 0.530 | 0.163 | 0 | 0.020 | 0.012 | 0 | 0 | 0 | 0 | 0 |
|  | 3***24** | **3** | 0.050 | 0 | 0.650 | 0.152 | 0.190 | 0.030 | 0 | 0 | 0.010 | 0 | 0 | 0 |
|  |  | **5** | 0.070 | 0 | 0.730 | 0.070 | 0.010 | 0.010 | 0 | 0 | 0 | 0 | 0 | 0 |
|  |  | **7** | 0.490 | 0.060 | 1 | 0.480 | 0.160 | 0.340 | 0 | 0 | 0.010 | 0 | 0 | 0 |
| ***500** | 3***12** | **3** | 0.270 | 0.020 | 0.920 | 0.250 | 0.540 | 0.020 | 0 | 0 | 0.040 | 0.011 | 0 | 0 |
|  |  | **5** | 0.250 | 0.020 | 0.860 | 0.210 | 0.100 | 0.010 | 0 | 0 | 0.010 | 0 | 0 | 0 |
|  |  | **7** | 0.310 | 0.030 | 0.940 | 0.270 | 0.120 | 0 | 0 | 0 | 0.040 | 0.011 | 0 | 0 |
|  | 3***24** | **3** | 0.220 | 0.010 | 0.960 | 0.260 | 0.690 | 0 | 0 | 0 | 0 | 0.010 | 0 | 0 |
|  |  | **5** | 0.850 | 0.770 | 1 | 0.840 | 0.960 | 0.010 | 0 | 0 | 0 | 0 | 0 | 0 |
|  |  | **7** | 0.880 | 0.700 | 1 | 0.890 | 0.910 | 0.040 | 0.010 | 0 | 0 | 0 | 0 | 0 |

[flushleft] Note. CHull = convex hull based on the Common Part Accounted For (CAF) index; BIC = Bayesian Information Criterion; PA = parallel analysis.

Table 8: Model selection TPR for unidimensional scales in function of large ARS and the simulated conditions

!

|  |  |  | **Model selection unidimensional scales with large ARS** | | | | | | | | | | | |
| --- | --- | --- | --- | --- | --- | --- | --- | --- | --- | --- | --- | --- | --- | --- |
|  |  |  | **Balanced Scales** | | | | | | **Unbalanced scales** | | | | | |
|  |  |  | **Pearson** | | | **Polychoric** | | | **Pearson** | | | **Polychoric** | | |
| **N** | **J** | **C** | **CHull** | **BIC** | **PA** | **CHull** | **BIC** | **PA** | **CHull** | **BIC** | **PA** | **CHull** | **BIC** | **PA** |
| ***250** | 3***12** | **3** | 0.660 | 0.780 | 0.970 | 0.540 | 0.960 | 0.990 | 0.023 | 0 | 0.070 | 0.069 | 0 | 0 |
|  |  | **5** | 0.740 | 0.940 | 0.980 | 0.730 | 0.990 | 0.990 | 0.011 | 0 | 0.010 | 0.035 | 0 | 0 |
|  |  | **7** | 0.970 | 0.990 | 0.990 | 0.960 | 1 | 1 | 0.012 | 0 | 0 | 0.012 | 0 | 0 |
|  | 3***24** | **3** | 0.950 | 1 | 1 | 0.830 | 1 | 1 | 0 | 0 | 0 | 0.010 | 0 | 0 |
|  |  | **5** | 0.940 | 1 | 1 | 0.890 | 1 | 1 | 0 | 0 | 0 | 0.010 | 0 | 0 |
|  |  | **7** | 0.970 | 1 | 1 | 0.950 | 1 | 1 | 0 | 0 | 0 | 0 | 0 | 0 |
| ***500** | 3***12** | **3** | 0.780 | 0.990 | 1 | 0.730 | 1 | 0.980 | 0 | 0 | 0.020 | 0.012 | 0.010 | 0 |
|  |  | **5** | 0.870 | 1 | 1 | 0.850 | 1 | 1 | 0 | 0 | 0.020 | 0 | 0 | 0 |
|  |  | **7** | 0.930 | 1 | 1 | 0.920 | 1 | 1 | 0.011 | 0 | 0.020 | 0 | 0 | 0 |
|  | 3***24** | **3** | 0.930 | 1 | 1 | 0.890 | 1 | 1 | 0 | 0 | 0.010 | 0 | 0 | 0 |
|  |  | **5** | 0.980 | 1 | 1 | 0.980 | 1 | 1 | 0 | 0 | 0 | 0 | 0 | 0 |
|  |  | **7** | 1 | 1 | 1 | 1 | 1 | 1 | 0 | 0 | 0 | 0 | 0 | 0 |

[flushleft] Note. CHull = convex hull based on the Common Part Accounted For (CAF) index; BIC = Bayesian Information Criterion; PA = parallel analysis.

Table 9: Model selection TPR for multidimensional scales in function of small ARS and the simulated conditions

!

|  |  |  | **Model selection multidimensional scales with small ARS** | | | | | | | | | | | |
| --- | --- | --- | --- | --- | --- | --- | --- | --- | --- | --- | --- | --- | --- | --- |
|  |  |  | **Balanced Scales** | | | | | | **Unbalanced scales** | | | | | |
|  |  |  | **Pearson** | | | **Polychoric** | | | **Pearson** | | | **Polychoric** | | |
| **N** | **J** | **C** | **CHull** | **BIC** | **PA** | **CHull** | **BIC** | **PA** | **CHull** | **BIC** | **PA** | **CHull** | **BIC** | **PA** |
| ***250** | 3***12** | **3** | 0.100 | 0 | 0.050 | 0.050 | 0 | 0 | 0.040 | 0 | 0.020 | 0.030 | 0 | 0 |
|  |  | **5** | 0.090 | 0 | 0.030 | 0.060 | 0 | 0 | 0.040 | 0 | 0 | 0.040 | 0 | 0 |
|  |  | **7** | 0.050 | 0 | 0.070 | 0.050 | 0 | 0 | 0.050 | 0 | 0.020 | 0.020 | 0 | 0 |
|  | 3***24** | **3** | 0.050 | 0 | 0.020 | 0.030 | 0 | 0 | 0.010 | 0 | 0.010 | 0.030 | 0 | 0 |
|  |  | **5** | 0.030 | 0 | 0 | 0.010 | 0 | 0 | 0.020 | 0 | 0 | 0.010 | 0 | 0 |
|  |  | **7** | 0.020 | 0 | 0.010 | 0.030 | 0 | 0 | 0.030 | 0 | 0.010 | 0.040 | 0 | 0 |
| ***500** | 3***12** | **3** | 0.020 | 0 | 0 | 0.020 | 0 | 0 | 0.040 | 0 | 0.040 | 0.050 | 0.020 | 0 |
|  |  | **5** | 0.050 | 0 | 0.040 | 0.070 | 0 | 0 | 0.030 | 0 | 0 | 0 | 0 | 0 |
|  |  | **7** | 0.080 | 0 | 0.060 | 0.050 | 0 | 0 | 0.030 | 0 | 0 | 0.020 | 0 | 0 |
|  | 3***24** | **3** | 0.020 | 0 | 0.020 | 0.040 | 0 | 0 | 0 | 0 | 0 | 0 | 0 | 0 |
|  |  | **5** | 0.040 | 0 | 0.070 | 0.060 | 0 | 0 | 0 | 0 | 0 | 0 | 0 | 0 |
|  |  | **7** | 0.060 | 0 | 0.160 | 0.040 | 0 | 0 | 0 | 0 | 0 | 0.010 | 0 | 0 |

[flushleft] Note. CHull = convex hull based on the Common Part Accounted For (CAF) index; BIC = Bayesian Information Criterion; PA = parallel analysis.

Table 10: Model selection TPR for multidimensional scales in function of medium ARS and the simulated conditions

!

|  |  |  | **Model selection multidimensional scales with medium ARS** | | | | | | | | | | | |
| --- | --- | --- | --- | --- | --- | --- | --- | --- | --- | --- | --- | --- | --- | --- |
|  |  |  | **Balanced Scales** | | | | | | **Unbalanced scales** | | | | | |
|  |  |  | **Pearson** | | | **Polychoric** | | | **Pearson** | | | **Polychoric** | | |
| **N** | **J** | **C** | **CHull** | **BIC** | **PA** | **CHull** | **BIC** | **PA** | **CHull** | **BIC** | **PA** | **CHull** | **BIC** | **PA** |
| ***250** | 3***12** | **3** | 0.370 | 0 | 0.460 | 0.290 | 0.210 | 0.170 | 0.050 | 0 | 0.010 | 0.060 | 0.010 | 0 |
|  |  | **5** | 0.310 | 0 | 0.560 | 0.320 | 0.010 | 0.060 | 0.070 | 0 | 0.020 | 0.040 | 0 | 0 |
|  |  | **7** | 0.420 | 0 | 0.570 | 0.450 | 0 | 0.060 | 0.070 | 0 | 0.020 | 0.060 | 0 | 0 |
|  | 3***24** | **3** | 0.550 | 0 | 0.850 | 0.520 | 0.430 | 0.360 | 0.040 | 0 | 0 | 0 | 0 | 0 |
|  |  | **5** | 0.660 | 0 | 0.910 | 0.690 | 0.290 | 0.250 | 0.010 | 0 | 0 | 0.010 | 0 | 0 |
|  |  | **7** | 0.620 | 0.010 | 0.960 | 0.660 | 0.090 | 0.160 | 0.020 | 0 | 0 | 0.020 | 0 | 0 |
| ***500** | 3***12** | **3** | 0.670 | 0.050 | 0.970 | 0.550 | 0.750 | 0.140 | 0.030 | 0 | 0.010 | 0.020 | 0 | 0 |
|  |  | **5** | 0.540 | 0.010 | 0.780 | 0.560 | 0.130 | 0.010 | 0.030 | 0 | 0 | 0.020 | 0 | 0 |
|  |  | **7** | 0.790 | 0.340 | 1 | 0.760 | 0.580 | 0.060 | 0.010 | 0 | 0 | 0.030 | 0 | 0 |
|  | 3***24** | **3** | 0.890 | 0.180 | 0.990 | 0.820 | 0.980 | 0.160 | 0.020 | 0 | 0 | 0.010 | 0 | 0 |
|  |  | **5** | 0.930 | 0.790 | 1 | 0.880 | 0.970 | 0.100 | 0.010 | 0 | 0 | 0.010 | 0 | 0 |
|  |  | **7** | 0.910 | 0.730 | 1 | 0.890 | 0.890 | 0 | 0 | 0 | 0 | 0 | 0 | 0 |

[flushleft] Note. CHull = convex hull based on the Common Part Accounted For (CAF) index; BIC = Bayesian Information Criterion; PA = parallel analysis.

Table 11: Model selection TPR for multidimensional scales in function of large ARS and the simulated conditions

!

|  |  |  | **Model selection multidimensional scales with large ARS** | | | | | | | | | | | |
| --- | --- | --- | --- | --- | --- | --- | --- | --- | --- | --- | --- | --- | --- | --- |
|  |  |  | **Balanced Scales** | | | | | | **Unbalanced scales** | | | | | |
|  |  |  | **Pearson** | | | **Polychoric** | | | **Pearson** | | | **Polychoric** | | |
| **N** | **J** | **C** | **CHull** | **BIC** | **PA** | **CHull** | **BIC** | **PA** | **CHull** | **BIC** | **PA** | **CHull** | **BIC** | **PA** |
| ***250** | 3***12** | **3** | 0.990 | 0.750 | 1 | 0.980 | 1 | 1 | 0.040 | 0 | 0.010 | 0.050 | 0.020 | 0 |
|  |  | **5** | 0.980 | 0.690 | 0.990 | 0.970 | 0.940 | 0.980 | 0.030 | 0 | 0.010 | 0.020 | 0 | 0 |
|  |  | **7** | 1 | 1 | 1 | 1 | 1 | 1 | 0.010 | 0 | 0 | 0.020 | 0 | 0 |
|  | 3***24** | **3** | 1 | 1 | 1 | 1 | 1 | 1 | 0.010 | 0 | 0 | 0.010 | 0 | 0 |
|  |  | **5** | 1 | 1 | 1 | 1 | 1 | 1 | 0.010 | 0 | 0 | 0 | 0 | 0 |
|  |  | **7** | 1 | 1 | 1 | 1 | 1 | 1 | 0.020 | 0 | 0 | 0 | 0 | 0 |
| ***500** | 3***12** | **3** | 1 | 1 | 1 | 1 | 1 | 1 | 0.040 | 0 | 0 | 0.050 | 0 | 0 |
|  |  | **5** | 1 | 0.990 | 1 | 1 | 1 | 0.970 | 0.040 | 0 | 0 | 0.030 | 0 | 0 |
|  |  | **7** | 1 | 1 | 1 | 1 | 1 | 0.990 | 0.020 | 0 | 0 | 0.030 | 0 | 0 |
|  | 3***24** | **3** | 1 | 1 | 1 | 1 | 1 | 1 | 0 | 0 | 0 | 0 | 0 | 0 |
|  |  | **5** | 1 | 1 | 1 | 1 | 1 | 1 | 0.010 | 0 | 0 | 0.010 | 0 | 0 |
|  |  | **7** | 1 | 1 | 1 | 1 | 1 | 1 | 0.010 | 0 | 0 | 0.010 | 0 | 0 |

[flushleft] Note. CHull = convex hull based on the Common Part Accounted For (CAF) index; BIC = Bayesian Information Criterion; PA = parallel analysis.

Table 12: $RMSE_{loadingsC}$ in unidimensional unbalanced scales when the ARS factor is extracted in function of the simulated conditions

!

|  |  |  | **Unidimensional unbalanced scales - $RMSE_{loadingsC}$ with ARS factor** | | | | | | | | | | | | | | | | |
| --- | --- | --- | --- | --- | --- | --- | --- | --- | --- | --- | --- | --- | --- | --- | --- | --- | --- | --- | --- |
|  |  |  | **Pearson** | | | | | | | | | **Polychoric** | | | | | | | |
|  |  |  | **Small ARS** | |  | **Medium ARS** | |  | **Large ARS** | |  | **Small ARS** | |  | **Medium ARS** | |  | **Large ARS** | |
| **N** | **J** | **C** | **Oblimin** | **FST** |  | **Oblimin** | **FST** |  | **Oblimin** | **FST** |  | **Oblimin** | **FST** |  | **Oblimin** | **FST** |  | **Oblimin** | **FST** |
| ***250** | 3***12** | **3** | 0.245 | 0.265 |  | 0.247 | 0.206 |  | 0.232 | 0.306 |  | 0.215 | 0.464 |  | 0.237 | 0.394 |  | 0.189 | 0.526 |
|  |  | **5** | 0.230 | 0.300 |  | 0.204 | 0.348 |  | 0.181 | 0.395 |  | 0.207 | 0.389 |  | 0.199 | 0.449 |  | 0.189 | 0.503 |
|  |  | **7** | 0.225 | 0.356 |  | 0.245 | 0.327 |  | 0.196 | 0.464 |  | 0.210 | 0.401 |  | 0.227 | 0.372 |  | 0.185 | 0.518 |
|  | 3***24** | **3** | 0.239 | 0.244 |  | 0.247 | 0.238 |  | 0.212 | 0.335 |  | 0.189 | 0.438 |  | 0.212 | 0.433 |  | 0.217 | 0.553 |
|  |  | **5** | 0.236 | 0.236 |  | 0.221 | 0.278 |  | 0.201 | 0.440 |  | 0.209 | 0.320 |  | 0.244 | 0.370 |  | 0.198 | 0.560 |
|  |  | **7** | 0.248 | 0.207 |  | 0.229 | 0.300 |  | 0.215 | 0.511 |  | 0.225 | 0.247 |  | 0.208 | 0.346 |  | 0.189 | 0.563 |
| ***500** | 3***12** | **3** | 0.245 | 0.255 |  | 0.220 | 0.261 |  | 0.209 | 0.357 |  | 0.228 | 0.451 |  | 0.189 | 0.462 |  | 0.199 | 0.587 |
|  |  | **5** | 0.237 | 0.311 |  | 0.214 | 0.408 |  | 0.199 | 0.440 |  | 0.212 | 0.401 |  | 0.219 | 0.516 |  | 0.191 | 0.553 |
|  |  | **7** | 0.200 | 0.394 |  | 0.236 | 0.344 |  | 0.198 | 0.436 |  | 0.196 | 0.441 |  | 0.218 | 0.389 |  | 0.191 | 0.490 |
|  | 3***24** | **3** | 0.249 | 0.259 |  | 0.240 | 0.297 |  | 0.225 | 0.328 |  | 0.198 | 0.459 |  | 0.194 | 0.506 |  | 0.207 | 0.547 |
|  |  | **5** | 0.259 | 0.279 |  | 0.240 | 0.321 |  | 0.219 | 0.363 |  | 0.229 | 0.368 |  | 0.226 | 0.414 |  | 0.201 | 0.467 |
|  |  | **7** | 0.247 | 0.265 |  | 0.209 | 0.362 |  | 0.213 | 0.407 |  | 0.242 | 0.308 |  | 0.238 | 0.407 |  | 0.202 | 0.456 |

[flushleft] Note. FST = fully-specified target.

Table 13: $RMSE_{loadingsC}$ in multidimensional unbalanced scales when the ARS factor is extracted in function of the simulated conditions

!

|  | |  | |  | | **Multidimensional unbalanced scales - $RMSE_{loadingsC}$ with ARS factor** |  |  |  |  |  |  |  |  |  |  |  |  |  |  |  |  |  |  |  |  |  |  |  |  |  |  |  |  |  |  |
| --- | --- | --- | --- | --- | --- | --- | --- | --- | --- | --- | --- | --- | --- | --- | --- | --- | --- | --- | --- | --- | --- | --- | --- | --- | --- | --- | --- | --- | --- | --- | --- | --- | --- | --- | --- | --- |
|  | |  | |  | | **Pearson** | | | | | | | | | | | | | | | | | | |  | | **Polychoric** |  |  |  |  |  |  |  |  |  |
|  | |  | |  | | **Small ARS** | | | | |  | | **Medium ARS** | | | | |  | | **Large ARS** | | | | |  | | **Small ARS** | | | | |  | | **Medium ARS** |  | **Large ARS** |
| **N** | **J** | | **C** | | **Oblimin** | | **FST** | **SST** |  | **Oblimin** | | **FST** | | **SST** |  | **Oblimin** | **FST** | | **SST** | |  | **Oblimin** | **FST** | **SST** | |  | | **Oblimin** | **FST** | **SST** |  | | **Oblimin** | | **FST** | **SST** |
| ***250** | 3***12** | | **3** | | 0.072 | | 0.327 | 0.047 |  | 0.073 | | 0.132 | | 0.047 |  | 0.047 | 0.047 | | 0.027 | |  | 0.034 | 0.122 | 0.011 | |  | | 0.033 | 0.030 | 0.012 |  | | 0.015 | | 0.096 | 0.029 |
|  |  | | **5** | | 0.046 | | 0.017 | 0.022 |  | 0.046 | | 0.025 | | 0.030 |  | 0.036 | 0.054 | | 0.016 | |  | 0.029 | 0.026 | 0.012 | |  | | 0.029 | 0.044 | 0.009 |  | | 0.017 | | 0.078 | 0.013 |
|  |  | | **7** | | 0.053 | | 0.013 | 0.028 |  | 0.041 | | 0.024 | | 0.024 |  | 0.021 | 0.068 | | 0.017 | |  | 0.043 | 0.016 | 0.024 | |  | | 0.036 | 0.031 | 0.023 |  | | 0.015 | | 0.082 | 0.023 |
|  | 3***24** | | **3** | | 0.049 | | 0.389 | 0.036 |  | 0.047 | | 0.204 | | 0.033 |  | 0.030 | 0.053 | | 0.022 | |  | 0.012 | 0.033 | 0.010 | |  | | 0.010 | 0.028 | 0.013 |  | | 0.028 | | 0.094 | 0.032 |
|  |  | | **5** | | 0.054 | | 0.322 | 0.041 |  | 0.025 | | 0.030 | | 0.016 |  | 0.016 | 0.063 | | 0.019 | |  | 0.035 | 0.054 | 0.022 | |  | | 0.010 | 0.043 | 0.014 |  | | 0.037 | | 0.093 | 0.043 |
|  |  | | **7** | | 0.057 | | 0.454 | 0.039 |  | 0.032 | | 0.011 | | 0.017 |  | 0.016 | 0.070 | | 0.020 | |  | 0.049 | 0.396 | 0.032 | |  | | 0.021 | 0.009 | 0.012 |  | | 0.022 | | 0.083 | 0.029 |
| ***500** | 3***12** | | **3** | | 0.071 | | 0.292 | 0.050 |  | 0.060 | | 0.026 | | 0.044 |  | 0.053 | 0.034 | | 0.031 | |  | 0.037 | 0.019 | 0.012 | |  | | 0.023 | 0.039 | 0.006 |  | | 0.014 | | 0.071 | 0.015 |
|  |  | | **5** | | 0.042 | | 0.013 | 0.026 |  | 0.033 | | 0.022 | | 0.017 |  | 0.025 | 0.048 | | 0.009 | |  | 0.027 | 0.023 | 0.011 | |  | | 0.018 | 0.036 | 0.014 |  | | 0.008 | | 0.073 | 0.018 |
|  |  | | **7** | | 0.033 | | 0.013 | 0.017 |  | 0.042 | | 0.010 | | 0.019 |  | 0.014 | 0.050 | | 0.007 | |  | 0.025 | 0.022 | 0.008 | |  | | 0.037 | 0.014 | 0.011 |  | | 0.007 | | 0.062 | 0.012 |
|  | 3***24** | | **3** | | 0.036 | | 0.026 | 0.029 |  | 0.037 | | 0.022 | | 0.030 |  | 0.024 | 0.038 | | 0.018 | |  | 0.013 | 0.029 | 0.019 | |  | | 0.009 | 0.026 | 0.015 |  | | 0.027 | | 0.073 | 0.032 |
|  |  | | **5** | | 0.026 | | 0.022 | 0.019 |  | 0.025 | | 0.021 | | 0.019 |  | 0.011 | 0.009 | | 0.007 | |  | 0.008 | 0.012 | 0.004 | |  | | 0.008 | 0.019 | 0.005 |  | | 0.014 | | 0.030 | 0.020 |
|  |  | | **7** | | 0.026 | | 0.022 | 0.021 |  | 0.014 | | 0.009 | | 0.010 |  | 0.009 | 0.045 | | 0.006 | |  | 0.017 | 0.012 | 0.013 | |  | | 0.007 | 0.009 | 0.004 |  | | 0.010 | | 0.052 | 0.011 |

[flushleft] Note. FST = fully-specified target; SST = semi-specified target.

Table 14: $RMSE_{loadingsARS}$ in unidimensional balanced scales in function of the simulated conditions

!

|  |  |  | **Unidimensional balanced scales - $RMSE_{loadingsARS}$** | | | | | | | | | | | | | | | | |
| --- | --- | --- | --- | --- | --- | --- | --- | --- | --- | --- | --- | --- | --- | --- | --- | --- | --- | --- | --- |
|  |  |  | **Pearson** | | | | | | | | | **Polychoric** | | | | | | | |
|  |  |  | **Small ARS** | |  | **Medium ARS** | |  | **Large ARS** | |  | **Small ARS** | |  | **Medium ARS** | |  | **Large ARS** | |
| **N** | **J** | **C** | **Oblimin** | **FST** |  | **Oblimin** | **FST** |  | **Oblimin** | **FST** |  | **Oblimin** | **FST** |  | **Oblimin** | **FST** |  | **Oblimin** | **FST** |
| ***250** | 3***12** | **3** | 0.149 | 0.030 |  | 0.078 | 0.035 |  | 0.012 | 0.059 |  | 0.196 | 0.045 |  | 0.125 | 0.023 |  | 0.012 | 0.059 |
|  |  | **5** | 0.162 | 0.033 |  | 0.088 | 0.034 |  | 0.032 | 0.038 |  | 0.171 | 0.039 |  | 0.098 | 0.029 |  | 0.032 | 0.038 |
|  |  | **7** | 0.153 | 0.027 |  | 0.083 | 0.033 |  | 0.011 | 0.018 |  | 0.176 | 0.030 |  | 0.101 | 0.029 |  | 0.011 | 0.018 |
|  | 3***24** | **3** | 0.147 | 0.014 |  | 0.086 | 0.054 |  | 0.027 | 0.051 |  | 0.145 | 0.021 |  | 0.121 | 0.039 |  | 0.027 | 0.051 |
|  |  | **5** | 0.161 | 0.017 |  | 0.082 | 0.049 |  | 0.021 | 0.047 |  | 0.165 | 0.016 |  | 0.106 | 0.042 |  | 0.021 | 0.047 |
|  |  | **7** | 0.150 | 0.014 |  | 0.074 | 0.019 |  | 0.048 | 0.025 |  | 0.157 | 0.015 |  | 0.084 | 0.015 |  | 0.048 | 0.025 |
| ***500** | 3***12** | **3** | 0.128 | 0.014 |  | 0.069 | 0.034 |  | 0.044 | 0.066 |  | 0.152 | 0.022 |  | 0.093 | 0.019 |  | 0.044 | 0.066 |
|  |  | **5** | 0.160 | 0.015 |  | 0.074 | 0.041 |  | 0.026 | 0.044 |  | 0.177 | 0.018 |  | 0.078 | 0.033 |  | 0.026 | 0.044 |
|  |  | **7** | 0.147 | 0.014 |  | 0.080 | 0.035 |  | 0.018 | 0.040 |  | 0.157 | 0.015 |  | 0.086 | 0.031 |  | 0.018 | 0.040 |
|  | 3***24** | **3** | 0.129 | 0.023 |  | 0.050 | 0.057 |  | 0.030 | 0.065 |  | 0.162 | 0.015 |  | 0.093 | 0.043 |  | 0.030 | 0.065 |
|  |  | **5** | 0.136 | 0.024 |  | 0.069 | 0.035 |  | 0.023 | 0.046 |  | 0.160 | 0.021 |  | 0.087 | 0.030 |  | 0.023 | 0.046 |
|  |  | **7** | 0.142 | 0.021 |  | 0.069 | 0.025 |  | 0.032 | 0.040 |  | 0.150 | 0.019 |  | 0.077 | 0.020 |  | 0.032 | 0.040 |

[flushleft] Note. FST = fully-specified target.

Table 15: $RMSE_{loadingsARS}$ in unidimensional unbalanced scales in function of the simulated conditions

!

|  |  |  | **Unidimensional unbalanced scales - $RMSE_{loadingsARS}$** | | | | | | | | | | | | | | | | |
| --- | --- | --- | --- | --- | --- | --- | --- | --- | --- | --- | --- | --- | --- | --- | --- | --- | --- | --- | --- |
|  |  |  | **Pearson** | | | | | | | | | **Polychoric** | | | | | | | |
|  |  |  | **Small ARS** | |  | **Medium ARS** | |  | **Large ARS** | |  | **Small ARS** | |  | **Medium ARS** | |  | **Large ARS** | |
| **N** | **J** | **C** | **Oblimin** | **FST** |  | **Oblimin** | **FST** |  | **Oblimin** | **FST** |  | **Oblimin** | **FST** |  | **Oblimin** | **FST** |  | **Oblimin** | **FST** |
| ***250** | 3***12** | **3** | 0.170 | 0.642 |  | 0.084 | 0.512 |  | 0.026 | 0.515 |  | 0.209 | 0.842 |  | 0.141 | 0.700 |  | 0.036 | 0.736 |
|  |  | **5** | 0.164 | 0.678 |  | 0.081 | 0.654 |  | 0.030 | 0.606 |  | 0.171 | 0.767 |  | 0.111 | 0.755 |  | 0.023 | 0.713 |
|  |  | **7** | 0.176 | 0.735 |  | 0.113 | 0.633 |  | 0.020 | 0.675 |  | 0.176 | 0.779 |  | 0.111 | 0.678 |  | 0.023 | 0.729 |
|  | 3***24** | **3** | 0.149 | 0.621 |  | 0.083 | 0.544 |  | 0.028 | 0.546 |  | 0.167 | 0.817 |  | 0.117 | 0.739 |  | 0.058 | 0.763 |
|  |  | **5** | 0.143 | 0.615 |  | 0.070 | 0.584 |  | 0.018 | 0.650 |  | 0.148 | 0.698 |  | 0.125 | 0.676 |  | 0.041 | 0.770 |
|  |  | **7** | 0.147 | 0.586 |  | 0.084 | 0.606 |  | 0.037 | 0.721 |  | 0.140 | 0.626 |  | 0.079 | 0.652 |  | 0.029 | 0.774 |
| ***500** | 3***12** | **3** | 0.152 | 0.634 |  | 0.056 | 0.568 |  | 0.028 | 0.568 |  | 0.201 | 0.830 |  | 0.094 | 0.769 |  | 0.041 | 0.797 |
|  |  | **5** | 0.159 | 0.690 |  | 0.093 | 0.714 |  | 0.020 | 0.651 |  | 0.165 | 0.780 |  | 0.132 | 0.822 |  | 0.028 | 0.764 |
|  |  | **7** | 0.146 | 0.772 |  | 0.095 | 0.651 |  | 0.025 | 0.647 |  | 0.157 | 0.819 |  | 0.092 | 0.695 |  | 0.018 | 0.700 |
|  | 3***24** | **3** | 0.151 | 0.638 |  | 0.082 | 0.603 |  | 0.023 | 0.538 |  | 0.169 | 0.837 |  | 0.106 | 0.812 |  | 0.040 | 0.758 |
|  |  | **5** | 0.167 | 0.657 |  | 0.091 | 0.627 |  | 0.020 | 0.573 |  | 0.169 | 0.747 |  | 0.108 | 0.721 |  | 0.013 | 0.677 |
|  |  | **7** | 0.151 | 0.644 |  | 0.075 | 0.668 |  | 0.014 | 0.618 |  | 0.160 | 0.687 |  | 0.117 | 0.714 |  | 0.017 | 0.666 |

[flushleft] Note. FST = fully-specified target.

Table 16: $RMSE_{loadingsARS}$ in multidimensional balanced scales in function of the simulated conditions

!

|  | |  | |  | | **Multidimensional balanced scales - $RMSE_{loadingsARS}$** |  |  |  |  |  |  |  |  |  |  |  |  |  |  |  |  |  |  |  |  |  |  |  |  |  |  |  |  |  |  |
| --- | --- | --- | --- | --- | --- | --- | --- | --- | --- | --- | --- | --- | --- | --- | --- | --- | --- | --- | --- | --- | --- | --- | --- | --- | --- | --- | --- | --- | --- | --- | --- | --- | --- | --- | --- | --- |
|  | |  | |  | | **Pearson** | | | | | | | | | | | | | | | | | | |  | | **Polychoric** |  |  |  |  |  |  |  |  |  |
|  | |  | |  | | **Small ARS** | | | | |  | | **Medium ARS** | | | | |  | | **Large ARS** | | | | |  | | **Small ARS** | | | | |  | | **Medium ARS** |  | **Large ARS** |
| **N** | **J** | | **C** | | **Oblimin** | | **FST** | **SST** |  | **Oblimin** | | **FST** | | **SST** |  | **Oblimin** | **FST** | | **SST** | |  | **Oblimin** | **FST** | **SST** | |  | | **Oblimin** | **FST** | **SST** |  | | **Oblimin** | | **FST** | **SST** |
| ***250** | 3***12** | | **3** | | 0.127 | | 0.061 | 0.329 |  | 0.180 | | 0.029 | | 0.379 |  | 0.237 | 0.033 | | 0.440 | |  | 0.143 | 0.062 | 0.369 | |  | | 0.198 | 0.017 | 0.414 |  | | 0.236 | | 0.018 | 0.473 |
|  |  | | **5** | | 0.128 | | 0.024 | 0.361 |  | 0.175 | | 0.038 | | 0.381 |  | 0.232 | 0.042 | | 0.432 | |  | 0.124 | 0.021 | 0.380 | |  | | 0.189 | 0.038 | 0.393 |  | | 0.254 | | 0.027 | 0.447 |
|  |  | | **7** | | 0.121 | | 0.031 | 0.344 |  | 0.149 | | 0.035 | | 0.392 |  | 0.219 | 0.017 | | 0.446 | |  | 0.128 | 0.027 | 0.351 | |  | | 0.157 | 0.034 | 0.400 |  | | 0.225 | | 0.016 | 0.452 |
|  | 3***24** | | **3** | | 0.109 | | 0.058 | 0.335 |  | 0.134 | | 0.047 | | 0.372 |  | 0.231 | 0.047 | | 0.421 | |  | 0.106 | 0.057 | 0.373 | |  | | 0.123 | 0.042 | 0.407 |  | | 0.218 | | 0.020 | 0.450 |
|  |  | | **5** | | 0.108 | | 0.076 | 0.348 |  | 0.141 | | 0.029 | | 0.385 |  | 0.241 | 0.044 | | 0.430 | |  | 0.103 | 0.074 | 0.366 | |  | | 0.143 | 0.019 | 0.403 |  | | 0.250 | | 0.029 | 0.444 |
|  |  | | **7** | | 0.118 | | 0.077 | 0.367 |  | 0.130 | | 0.035 | | 0.388 |  | 0.234 | 0.034 | | 0.441 | |  | 0.111 | 0.079 | 0.375 | |  | | 0.155 | 0.031 | 0.395 |  | | 0.218 | | 0.027 | 0.447 |
| ***500** | 3***12** | | **3** | | 0.136 | | 0.038 | 0.326 |  | 0.148 | | 0.046 | | 0.373 |  | 0.244 | 0.056 | | 0.418 | |  | 0.152 | 0.035 | 0.361 | |  | | 0.153 | 0.064 | 0.408 |  | | 0.219 | | 0.029 | 0.448 |
|  |  | | **5** | | 0.132 | | 0.026 | 0.356 |  | 0.157 | | 0.041 | | 0.375 |  | 0.209 | 0.055 | | 0.441 | |  | 0.135 | 0.023 | 0.374 | |  | | 0.148 | 0.033 | 0.391 |  | | 0.217 | | 0.042 | 0.457 |
|  |  | | **7** | | 0.132 | | 0.046 | 0.359 |  | 0.146 | | 0.041 | | 0.379 |  | 0.207 | 0.046 | | 0.441 | |  | 0.129 | 0.045 | 0.368 | |  | | 0.149 | 0.042 | 0.386 |  | | 0.243 | | 0.038 | 0.447 |
|  | 3***24** | | **3** | | 0.102 | | 0.057 | 0.339 |  | 0.151 | | 0.042 | | 0.364 |  | 0.212 | 0.061 | | 0.433 | |  | 0.103 | 0.051 | 0.375 | |  | | 0.124 | 0.031 | 0.397 |  | | 0.233 | | 0.035 | 0.464 |
|  |  | | **5** | | 0.106 | | 0.036 | 0.358 |  | 0.132 | | 0.029 | | 0.389 |  | 0.211 | 0.047 | | 0.438 | |  | 0.102 | 0.031 | 0.375 | |  | | 0.133 | 0.023 | 0.407 |  | | 0.198 | | 0.031 | 0.453 |
|  |  | | **7** | | 0.100 | | 0.037 | 0.359 |  | 0.140 | | 0.024 | | 0.387 |  | 0.253 | 0.025 | | 0.450 | |  | 0.103 | 0.037 | 0.367 | |  | | 0.142 | 0.019 | 0.394 |  | | 0.223 | | 0.016 | 0.457 |

Note. FST = fully-specified target; SST = semi-specified target.

Table 17: $RMSE_{loadingsARS}$ in multidimensional unbalanced scales in function of the simulated conditions

!

|  | |  | |  | | **Multidimensional unbalanced scales - $RMSE_{loadingsARS}$** |  |  |  |  |  |  |  |  |  |  |  |  |  |  |  |  |  |  |  |  |  |  |  |  |  |  |  |  |  |  |
| --- | --- | --- | --- | --- | --- | --- | --- | --- | --- | --- | --- | --- | --- | --- | --- | --- | --- | --- | --- | --- | --- | --- | --- | --- | --- | --- | --- | --- | --- | --- | --- | --- | --- | --- | --- | --- |
|  | |  | |  | | **Pearson** | | | | | | | | | | | | | | | | | | |  | | **Polychoric** |  |  |  |  |  |  |  |  |  |
|  | |  | |  | | **Small ARS** | | | | |  | | **Medium ARS** | | | | |  | | **Large ARS** | | | | |  | | **Small ARS** | | | | |  | | **Medium ARS** |  | **Large ARS** |
| **N** | **J** | | **C** | | **Oblimin** | | **FST** | **SST** |  | **Oblimin** | | **FST** | | **SST** |  | **Oblimin** | **FST** | | **SST** | |  | **Oblimin** | **FST** | **SST** | |  | | **Oblimin** | **FST** | **SST** |  | | **Oblimin** | | **FST** | **SST** |
| ***250** | 3***12** | | **3** | | 0.155 | | 0.836 | 0.238 |  | 0.223 | | 0.449 | | 0.231 |  | 0.347 | 0.197 | | 0.247 | |  | 0.180 | 0.485 | 0.275 | |  | | 0.237 | 0.271 | 0.262 |  | | 0.349 | | 0.287 | 0.278 |
|  |  | | **5** | | 0.168 | | 0.324 | 0.256 |  | 0.249 | | 0.262 | | 0.230 |  | 0.303 | 0.217 | | 0.247 | |  | 0.173 | 0.335 | 0.276 | |  | | 0.226 | 0.301 | 0.256 |  | | 0.321 | | 0.260 | 0.256 |
|  |  | | **7** | | 0.175 | | 0.319 | 0.261 |  | 0.224 | | 0.245 | | 0.230 |  | 0.323 | 0.245 | | 0.263 | |  | 0.149 | 0.311 | 0.266 | |  | | 0.219 | 0.260 | 0.239 |  | | 0.337 | | 0.266 | 0.275 |
|  | 3***24** | | **3** | | 0.139 | | 0.928 | 0.249 |  | 0.215 | | 0.560 | | 0.236 |  | 0.318 | 0.194 | | 0.253 | |  | 0.133 | 0.313 | 0.291 | |  | | 0.198 | 0.246 | 0.270 |  | | 0.312 | | 0.281 | 0.281 |
|  |  | | **5** | | 0.145 | | 0.822 | 0.249 |  | 0.214 | | 0.260 | | 0.245 |  | 0.303 | 0.232 | | 0.268 | |  | 0.132 | 0.393 | 0.265 | |  | | 0.210 | 0.298 | 0.260 |  | | 0.309 | | 0.280 | 0.287 |
|  |  | | **7** | | 0.153 | | 1.031 | 0.241 |  | 0.206 | | 0.244 | | 0.245 |  | 0.303 | 0.238 | | 0.259 | |  | 0.151 | 0.940 | 0.249 | |  | | 0.204 | 0.238 | 0.250 |  | | 0.306 | | 0.262 | 0.266 |
| ***500** | 3***12** | | **3** | | 0.151 | | 0.779 | 0.235 |  | 0.223 | | 0.234 | | 0.226 |  | 0.314 | 0.164 | | 0.236 | |  | 0.169 | 0.309 | 0.274 | |  | | 0.221 | 0.294 | 0.261 |  | | 0.317 | | 0.248 | 0.261 |
|  |  | | **5** | | 0.161 | | 0.299 | 0.253 |  | 0.223 | | 0.231 | | 0.254 |  | 0.315 | 0.208 | | 0.253 | |  | 0.163 | 0.325 | 0.270 | |  | | 0.229 | 0.258 | 0.274 |  | | 0.314 | | 0.251 | 0.265 |
|  |  | | **7** | | 0.170 | | 0.311 | 0.267 |  | 0.224 | | 0.238 | | 0.241 |  | 0.314 | 0.215 | | 0.259 | |  | 0.146 | 0.326 | 0.275 | |  | | 0.248 | 0.246 | 0.249 |  | | 0.326 | | 0.235 | 0.262 |
|  | 3***24** | | **3** | | 0.135 | | 0.283 | 0.248 |  | 0.206 | | 0.226 | | 0.231 |  | 0.300 | 0.165 | | 0.251 | |  | 0.120 | 0.338 | 0.286 | |  | | 0.196 | 0.266 | 0.266 |  | | 0.303 | | 0.249 | 0.278 |
|  |  | | **5** | | 0.132 | | 0.275 | 0.263 |  | 0.201 | | 0.225 | | 0.244 |  | 0.300 | 0.141 | | 0.256 | |  | 0.127 | 0.300 | 0.281 | |  | | 0.206 | 0.260 | 0.261 |  | | 0.292 | | 0.178 | 0.268 |
|  |  | | **7** | | 0.130 | | 0.275 | 0.264 |  | 0.210 | | 0.225 | | 0.252 |  | 0.299 | 0.192 | | 0.257 | |  | 0.132 | 0.289 | 0.273 | |  | | 0.202 | 0.239 | 0.259 |  | | 0.302 | | 0.210 | 0.263 |

Note. FST = fully-specified target; SST = semi-specified target.

Table 18: Main effects on MMAB for zero loadings in multidimensional unbalanced scales when the ARS factor is extracted in function of the simulated conditions

!

|  | **Multidimensional unbalanced scales - MMAB with ARS factor** | | | | | | | | | | | | | | | | | |
| --- | --- | --- | --- | --- | --- | --- | --- | --- | --- | --- | --- | --- | --- | --- | --- | --- | --- | --- |
|  | **Small ARS** | | | | | | **Medium ARS** | | | | | | **Large ARS** | | | | | |
|  | **Pearson** | | | **Polychoric** | | | **Pearson** | | | **Polychoric** | | | **Pearson** | | | **Polychoric** | | |
|  | **Oblimin** | **FST** | **SST** | **Oblimin** | **FST** | **SST** | **Oblimin** | **FST** | **SST** | **Oblimin** | **FST** | **SST** | **Oblimin** | **FST** | **SST** | **Oblimin** | **FST** | **SST** |
| **N = 250** | 0.171 | 0.532 | 0.120 | 0.182 | 0.398 | 0.128 | 0.171 | 0.324 | 0.124 | 0.181 | 0.281 | 0.131 | 0.169 | 0.277 | 0.119 | 0.178 | 0.297 | 0.125 |
| **N = 500** | 0.116 | 0.277 | 0.085 | 0.125 | 0.233 | 0.093 | 0.116 | 0.234 | 0.084 | 0.126 | 0.242 | 0.089 | 0.117 | 0.227 | 0.084 | 0.126 | 0.244 | 0.093 |
| **C = 3** | 0.148 | 0.511 | 0.104 | 0.168 | 0.310 | 0.116 | 0.151 | 0.350 | 0.105 | 0.168 | 0.283 | 0.117 | 0.151 | 0.253 | 0.105 | 0.170 | 0.290 | 0.120 |
| **C = 5** | 0.143 | 0.337 | 0.100 | 0.153 | 0.272 | 0.108 | 0.140 | 0.244 | 0.107 | 0.147 | 0.259 | 0.107 | 0.141 | 0.243 | 0.098 | 0.144 | 0.260 | 0.103 |
| **C = 7** | 0.139 | 0.366 | 0.105 | 0.140 | 0.364 | 0.108 | 0.140 | 0.241 | 0.100 | 0.146 | 0.243 | 0.105 | 0.137 | 0.259 | 0.101 | 0.142 | 0.262 | 0.105 |
| **J = 12** | 0.146 | 0.365 | 0.094 | 0.156 | 0.289 | 0.103 | 0.147 | 0.282 | 0.099 | 0.158 | 0.266 | 0.103 | 0.150 | 0.257 | 0.095 | 0.159 | 0.276 | 0.103 |
| **J = 24** | 0.141 | 0.445 | 0.111 | 0.151 | 0.343 | 0.118 | 0.140 | 0.275 | 0.109 | 0.150 | 0.257 | 0.117 | 0.135 | 0.246 | 0.107 | 0.145 | 0.266 | 0.115 |

[flushleft] Note. FST = fully-specified target; SST = semi-specified target.

Table 19: Main effects on $RMSE_{FactorCorr}$ in function of the strength of the ARS and the simulated conditions when ARS is extracted in unbalanced scales

!

|  | **$RMSE_{FactorCorr}$ with ARS factor** | | | | | | | | | | | | | | | | | |
| --- | --- | --- | --- | --- | --- | --- | --- | --- | --- | --- | --- | --- | --- | --- | --- | --- | --- | --- |
|  | **Small ARS** | | | | | | **Medium ARS** | | | | | | **Large ARS** | | | | | |
|  | **Pearson** | | | **Polychoric** | | | **Pearson** | | | **Polychoric** | | | **Pearson** | | | **Polychoric** | | |
|  | **Oblimin** | **FST** | **SST** | **Oblimin** | **FST** | **SST** | **Oblimin** | **FST** | **SST** | **Oblimin** | **FST** | **SST** | **Oblimin** | **FST** | **SST** | **Oblimin** | **FST** | **SST** |
| **N = 250** | 0.010 | 0.226 | 0.015 | 0.014 | 0.150 | 0.020 | 0.012 | 0.127 | 0.012 | 0.012 | 0.104 | 0.016 | 0.020 | 0.092 | 0.041 | 0.025 | 0.094 | 0.056 |
| **N = 500** | 0.014 | 0.121 | 0.006 | 0.002 | 0.100 | 0.006 | 0.016 | 0.100 | 0.013 | 0.021 | 0.098 | 0.021 | 0.016 | 0.089 | 0.028 | 0.014 | 0.090 | 0.040 |
| **C = 3** | 0.018 | 0.227 | 0.015 | 0.012 | 0.120 | 0.022 | 0.012 | 0.140 | 0.014 | 0.015 | 0.105 | 0.021 | 0.019 | 0.090 | 0.038 | 0.014 | 0.094 | 0.056 |
| **C = 5** | 0.004 | 0.138 | 0.007 | 0.002 | 0.110 | 0.009 | 0.013 | 0.098 | 0.012 | 0.016 | 0.098 | 0.020 | 0.016 | 0.092 | 0.037 | 0.012 | 0.092 | 0.042 |
| **C = 7** | 0.013 | 0.155 | 0.010 | 0.011 | 0.145 | 0.009 | 0.016 | 0.102 | 0.012 | 0.019 | 0.101 | 0.014 | 0.018 | 0.090 | 0.028 | 0.032 | 0.090 | 0.045 |
| **J = 12** | 0.016 | 0.168 | 0.012 | 0.011 | 0.122 | 0.017 | 0.016 | 0.120 | 0.014 | 0.016 | 0.110 | 0.017 | 0.026 | 0.100 | 0.042 | 0.027 | 0.101 | 0.049 |
| **J = 24** | 0.008 | 0.178 | 0.010 | 0.005 | 0.127 | 0.010 | 0.012 | 0.107 | 0.011 | 0.017 | 0.092 | 0.020 | 0.010 | 0.082 | 0.027 | 0.012 | 0.083 | 0.046 |

[flushleft] Note. FST = fully-specified target; SST = semi-specified target.
